# De Novo Assembly and Analysis of *Polygonatum sibiricum* Transcriptome and Identification of Genes Involved in Polysaccharide Biosynthesis

**DOI:** 10.3390/ijms18091950

**Published:** 2017-09-12

**Authors:** Shiqiang Wang, Bin Wang, Wenping Hua, Junfeng Niu, Kaikai Dang, Yi Qiang, Zhezhi Wang

**Affiliations:** 1 National Engineering Laboratory for Resource Developing of Endangered Chinese Crude Drugs in Northwest of China, Key Laboratory of the Ministry of Education for Medicinal Resources and Natural Pharmaceutical Chemistry, College of Life Sciences, Shaanxi Normal University, Xi’an 710119, Shaanxi, China; wsq@snnu.edu.cn (S.W.); wangb@ecit.cn (B.W.); niujunfeng6829@126.com (J.N.); kkdang@snnu.edu.cn (K.D.); yqiang@snnu.edu.cn (Y.Q.); 2 College of Chemistry, Biology and Materials Science, East China University of Technology, Nanchang 330013, Jiangxi, China; 3 College of Life Sciences and Food Engineering, Shaanxi XueQian Normal University, Xi’an 710119, Shaanxi, China; huawenping@126.com

**Keywords:** *Polygonatum sibiricum*, polysaccharide, transcriptome, polysaccharide biosynthesis, qRT-PCR

## Abstract

*Polygonatum sibiricum* polysaccharides (PSPs) are used to improve immunity, alleviate dryness, promote the secretion of fluids, and quench thirst. However, the PSP biosynthetic pathway is largely unknown. Understanding the genetic background will help delineate that pathway at the molecular level so that researchers can develop better conservation strategies. After comparing the PSP contents among several different *P. sibiricum* germplasms, we selected two groups with the largest contrasts in contents and subjected them to HiSeq2500 transcriptome sequencing to identify the candidate genes involved in PSP biosynthesis. In all, 20 kinds of enzyme-encoding genes were related to PSP biosynthesis. The polysaccharide content was positively correlated with the expression patterns of β-fructofuranosidase (*sacA*), fructokinase (*scrK*), UDP-glucose 4-epimerase (*GALE*), Mannose-1-phosphate guanylyltransferase (*GMPP*), and UDP-glucose 6-dehydrogenase (*UGDH*), but negatively correlated with the expression of Hexokinase (*HK*). Through qRT-PCR validation and comprehensive analysis, we determined that *sacA*, *HK*, and *GMPP* are key genes for enzymes within the PSP metabolic pathway in *P. sibiricum.* Our results provide a public transcriptome dataset for this species and an outline of pathways for the production of polysaccharides in medicinal plants. They also present more information about the PSP biosynthesis pathway at the molecular level in *P. sibiricum* and lay the foundation for subsequent research of gene functions.

## 1. Introduction

*Polygonatum sibiricum* is a well-known traditional Chinese herb that has been widely applied for hundreds of years to treat many diseases in China, Korea, Japan, and other East Asian countries [1]. Now, it has become endangered due to the uncontrolled excavation of natural resources [2]. In the Chinese Pharmacopoeia, “*P. sibiricum*” is prescribed as the dried rhizome of *Polygonatum sibiricum* Red., *Polygonatum cyrtonema* Hua, and *Polygonatum kingianum* Coll. et Hemsl. within the genus *Polygonatum*. Polysaccharides isolated from *P. sibiricum* (PSPs) are major contributors to sweet taste and have many important biological functions [3]. The results of animal and cell experiments have shown that these compounds can promote the proliferation and enhance the viability of mesenchymal stem cells in bones [4], help prevent Alzheimer’s disease [5] and osteoporosis [6], serve as hypolipidemics and antiatherosclerotics [7], and improve immunologic functions [8]. Their polysaccharide structures and composition are closely related to their biological functions. Therefore, it is important to understand the biosynthesis, metabolism, and regulation of PSPs in *P. sibiricum*.

A large volume of sequences relevant to the biosynthesis of several natural products has been identified via next-generation sequencing technology [9]. However, little research has been reported about polysaccharide biosynthesis. Using boiling water extractions and alcohol precipitation, the result demonstrated that PSPs are composed of fructose [10], but genetic information for genes related to their biosynthesis remains unknown.

In this study, we conducted a comparative analysis of the transcriptomes for two germplasms of *P. sibiricum* that contrast sharply in their PSP contents. After identifying candidate genes involved in PSP biosynthesis, we verified the quality of our dataset and confirmed these putative PSP genes through quantitative real-time PCR (qRT-PCR). Our results provide insight into the biosynthesis of polysaccharides in this species.

## 2. Results

### 2.1. PSP Content Varies among Polygonatum sibiricum Germplasms

**Figure 1:**
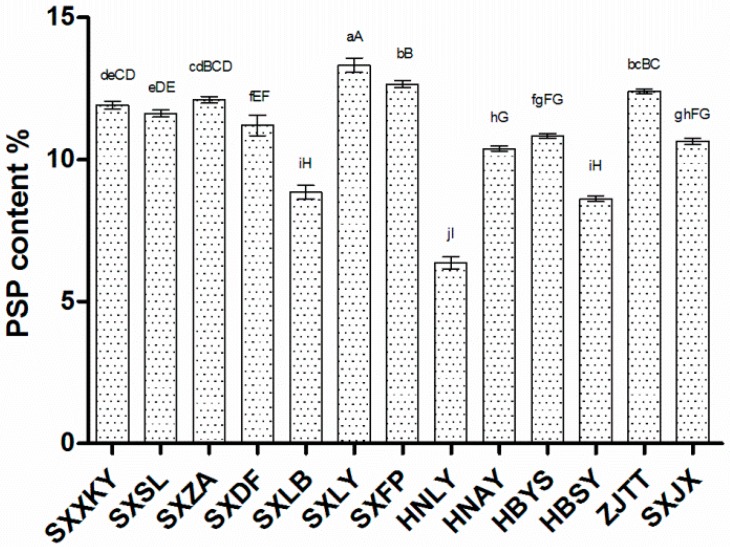
*Polygonatum sibiricum* polysaccharide (PSP) contents in different germplasms of *Polygonatum sibiricum*. SXXKY: Xiakou Yi, Shaanxi, China; SXSL: Shangluo, Shaanxi; SXZA: ZhenAn, Shaanxi; SXDF: Danfeng, Shaanxi; SXLB: Liuba, Shaanxi; SXLY: Lueyang, Shaanxi; SXFP: Foping, Shaanxi; HNLY: Luoyang, Henan, China; HNAY: Anyang, Henan; HBYS: Yingshan, Hubei, China; HBSY: Shiyan, Hubei; ZJTT: Tiantai, Zhejiang, China; and SXJX: Jiangxian, Shanxi, China. For the different germplasms, PSP content is significantly different between germplasms at *p* < 0.05 (different lowercase letters) and *p* < 0.01 (different capital letters).

Crude water-soluble polysaccharide was extracted from the dried rhizomes of different germplasms of *Polygonatum sibiricum*. As shown in Figure 1, the PSP content was the highest in Lueyang, Shaanxi (SXLY) (13.33%) and the lowest in Luoyang, Henan (HNLY) (6.37%). Because of this wide gap, we chose these two germplasms for transcriptome library preparation and RNA-sequencing (RNA-seq).

### 2.2. Illumina Sequencing, De Novo Assembly, and Assessment of Assembly Program

**Figure 2:**
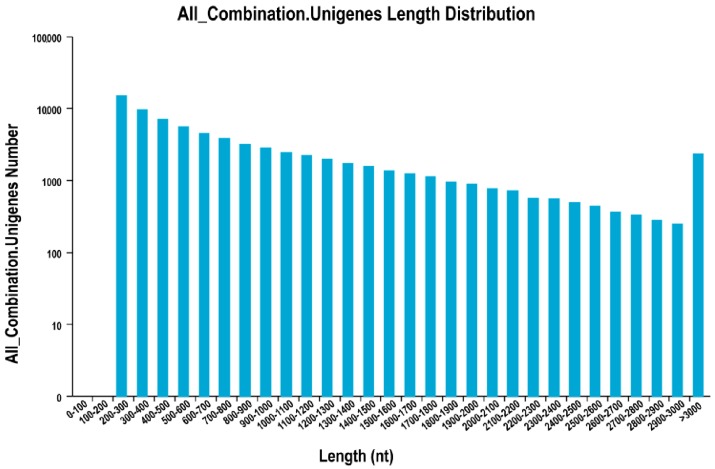
Distribution of unigene lengths from *Polygonatum sibiricum*.

We obtained 72.06 Gb of clean data from seven samples via the Illumina system. The mixed materials (PSABC) used to provide the genetic background comprised a pool of rhizomes, stems, and leaves. The PS01-03 samples represented the SXLY rhizomes, while the PS04-06 samples contained the HNLY rhizomes. Each sample had more than 6.79 Gb of clean data, with Q30 values greater than 93.31% (Table 1). After adaptor sequences, ambiguous reads, and low-quality reads were removed, the sequence data were assembled with Trinity software to produce 23,733,072 contigs, with an N50 of 45 bp. Based on paired-end information and the similarity of contig sequences, we obtained 133,121 transcripts, with an N50 of 1532 bp. Finally, 74,130 unigenes were generated with an N50 of 1364 bp. The size distribution was homogeneous (Figure 2); 30.36% of the unigenes were longer than 1000 bp, while 57.34% were longer than 500 bp (Table 2). These findings indicated that the assembly was successful [11].

### 2.3. Functional Annotations

All unigenes generated by HiSeq 2500 were aligned to information in the non-redundant (NR), Clusters of Orthologous Groups (COG), euKaryotic Orthologous Groups (KOG), Gene Ontology (GO), Swiss-Prot, Protein family (Pfam), and Kyoto Encyclopedia of Genes and Genomes (KEGG) protein databases by BLASTx and BLASTn. After all of the amino acid sequences were predicted, we compared them with the Pfam database to obtain annotation information. In total, 35,793 unigenes (48.28%) were aligned to homologous sequences in those public databases. Of them, 14,618 (19.72%) were 300 to 1000 bp long, while 17,140 (23.12%) were longer than 1000 bp (Table 3).

**Figure 3:**
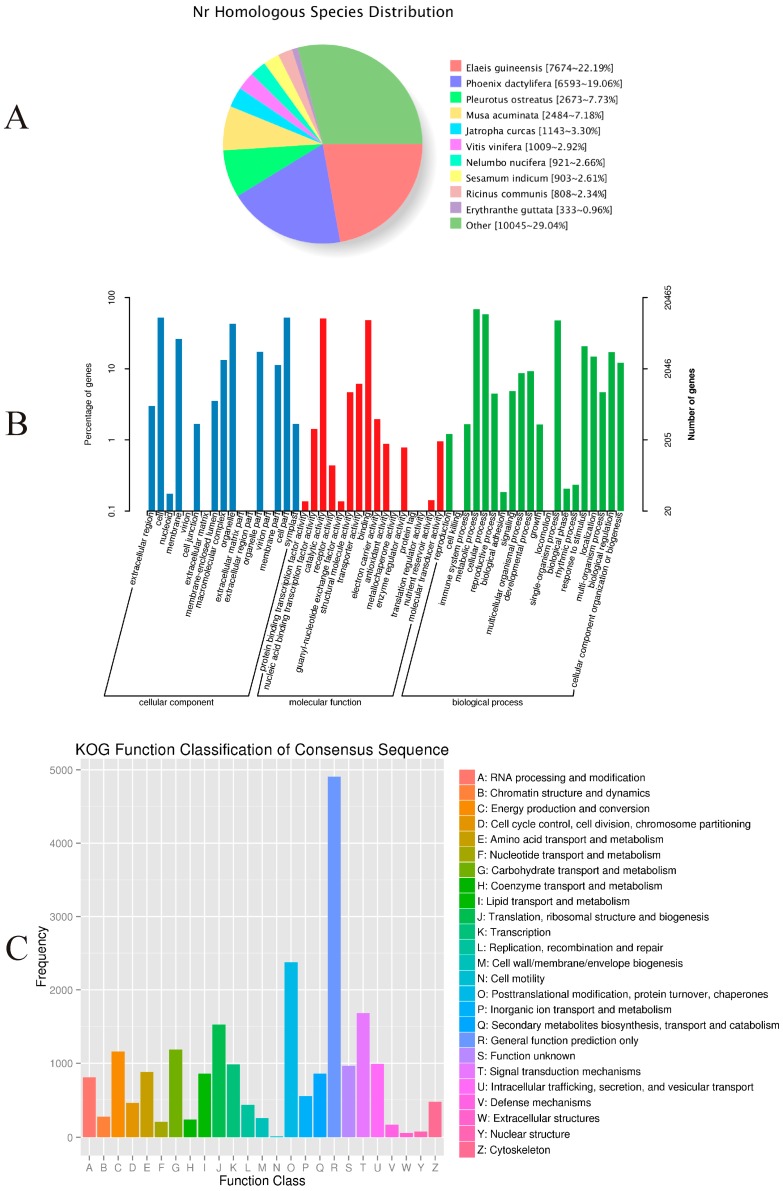
Functional annotations for *Polygonatum sibiricum*. (**A**) NR classifications of unigenes; (**B**) GO classifications derived via Solexa sequencing; (**C**) COG classifications derived via Solexa sequencing.

Approximately 46.68% (34,601) of the unigenes were annotated with reference to the NR database. As shown in Figure 3A, 22.19% had top matches to the sequences from *Elaeis guineensis*, 19.06% to the sequences from *Phoenix dactylifera*, and 7.73% to the sequences from *Pleurotus ostreatus*.

Among the 34,601 most significant BLASTx hits against the NR database, 20,465 unigenes could be assigned to one or more GO terms. These were assigned to 53 functional groups (Figure 3B). Among those categorized in “cellular components”, 10,797 (52.76%) were related to “cell part” (GO: 0044464), followed by “membrane” (GO: 0016020; 5395, or 26.36%). Within the molecular functions, the assignments were mostly enriched in “catalytic activity” (GO: 0003824; 10,444) and “binding” (GO: 0005488; 9905). The GO terms of biological processes were mainly grouped into “metabolic process” (GO: 0008152; 14,065) and “cellular process” (GO: 0009987; 11,945).

The COG assignments were used for further evaluation of the completeness of our *P. sibiricum* transcriptome library and the reliability of the annotation process. Overall, 12,175 (16.42%) unigenes were assigned to 25 COG categories (Figure 3C). Among these, the most numerous were unigenes belonging to the “General function prediction only” (2965, or 24.35% of the total), followed by “Replication, recombination, and repair” (1441, 11.83%), and “Translation, ribosomal structure, and biogenesis” (1426, 11.74%). Another 946 unigenes (7.77%) were assigned to “carbohydrate transport and metabolism”.

**Figure 4:**
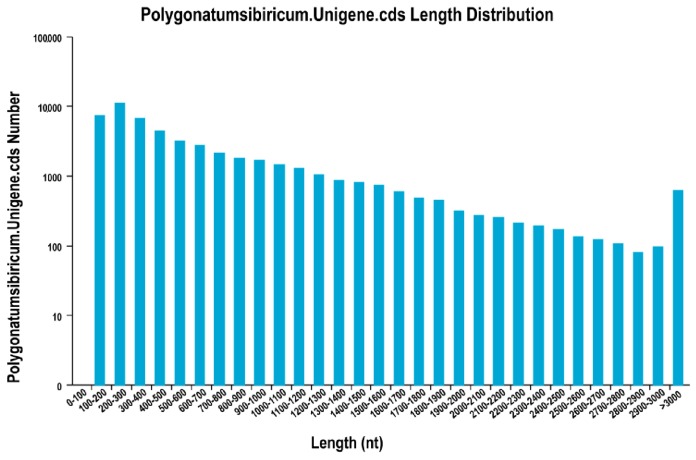
Distribution of lengths for coding sequences (CDSs) predicted from *Polygonatum sibiricum* unigenes.

Using the three public protein databases, we obtained 51,461 coding sequences (CDSs), which accounted for 69.42% of all unigenes identified here. The length distribution of CDSs (Figure 4) indicated that 33,862 were 200 to 1000 bp long, and that 34.20% were longer than 500 bp. Another 8014 (15.57%) were 1000 to 2000 bp long, 1636 (3.19%) were 2000 to 3000 bp long, and 629 (1.22%) were longer than 3000 bp.

### 2.4. Kyoto Encyclopedia of Genes and Genomes Pathway Analysis

To understand the biological processes in *P. sibiricum*, we mapped the unigenes annotated to the KEGG database and assigned 14,267 unigenes to 128 KEGG pathways (Table S1). Among them, 891 were mapped to ribosome pathways, 657 to “Carbon metabolism” pathways, 578 to pathways for “Biosynthesis of amino acids”, 473 for “Protein processing in endoplasmic reticulum”, and 284 for “Starch and sucrose metabolism” (Table 4). Furthermore, 1860 unigenes were mapped to the relevant pathways of polysaccharide biosynthesis, including glycolysis/gluconeogenesis, starch and sucrose metabolism, pyruvate metabolism, and amino sugar and nucleotide sugar metabolism (Table 5).

### 2.5. Candidate Genes Involved in PSP Biosynthesis

To understand the biosynthesis of PSP and to identify the responsible genes, we annotated the transcripts related to the KO00500 and KO00520 pathway. Mainly based on the KEGG database, we determined the key enzyme genes involved in these pathways (Table 6).

Plant polysaccharides are formed by the active nucleotide-diphospho-sugar (NDP-sugar) precursors, which are added to the residues of polysaccharides and glycoconjugates by the action of various glycosyltransferases (GTs) [12,13]. According to previous studies [12,13,14,15,16,17,18,19,20], PSP biosynthesis can be divided into three main stages. Firstly, sucrose is converted to Glc-1P and Fru-6P. During these processes, many enzymes play their roles, such as the β-fructofuranosidase (encoded by *sacA*) [14] that converts sucrose to Glc-6P and Fructose, and Phosphoglucomutase (encoded by *pgm*) that isomerizes Glc-6P to Glc-1P [15]. Hexokinase (encoded by *HK*) [16] and Fructokinase (encoded by *scrK*) [17] also take part in the biosynthesis of Fru-6P. Secondly, uridine diphosphate glucose (UDP-Glc) is derived from Glc-1P immediately [18], and Fru-6P is converted to GDP-Man indirectly [19]. Based on UDP-Glc and guanosine diphospho mannose (GDP-Man), other NDP sugars are further converted through the action of NDP-sugar interconversion enzymes (NSEs) [20], such as UDP-glucose 4-epimerase (*GALE*), UDP-d-galactose dehydrogenase (*UGD*), UDP-glucuronate 4-epimerase (*UGE*), UDP-glucose 6-dehydrogenase (*UGDH*), UDP-arabinose 4-epimerase (*UXE*), UDP-glucose 4,6-dehydratase (*RHM*), 3,5-epimerase-4-reductase (*UER1*), and GDP-mannose 4,6-dehydratase (*GMDS*). Finally, various NDP-sugars form growing polysaccharide chains by the action of glycosyltransferases (GTs).

In plants, UDP-Glc is a key precursor of nucleoside diphosphate (NDP)-sugars, and it is derived from 1-P-glucose (Glu-1-P) by the action of Uridine-diphosphate glucose pyrophosphorylase (encoded by *galU*) [18,21]. In the *P. sibiricum* library, Unigene 124,676 was identified as a homologue of *galU.* This 1153 bp unigene contained a complete open reading frame, and shared the highest amino acid identity (86%) with *galU* from *Ricinus communis* (Accession Number: XM_002526548.2) (Figures S1 and S2). Sugars produced by plants, such as the hexose sugars glucose and fructose, serve as the basic material for the production and accumulation of organic compounds in plants. The first step of hexose sugars’ metabolism is to phosphorylate them. Currently, only two kinds of plant enzymes, hexokinases (HKs) and fructokinases (scrKs), have been found to catalyze the conversion of glucose and fructose. Here, we found that the 2017 bp Unigene 137,389 was a *HK* homologue that shared the highest amino acid identity (72%) with *HK* from *Phoenix dactylifera* (Accession Number: XM_008797620.2) (Figures S1 and S3). Furthermore, the 1803 bp Unigene 107,236 was a homologue of *scrK* from *Elaeis guineensis* (Accession number: XM_010934340.2) (Figures S1 and S4).

An examination of the monosaccharide composition (Table S2) showed that PSPs are formed by the polymerization of NDP-sugars such as UDP-Glc, uridine diphosphate galactose (UDP-Gal), uridine diphosphate arabinose (UDP-Ara), uridine diphosphate rhamnose (UDP-Rha), GDP-Man, and guanosine diphosphate fucose (GDP-Fuc). UDP-Glc is derived from Glc-1P directly, and GDP-Man from Fru-6P indirectly, and the others are derived from UDP-Glc and GDP-Man through the actions of NSEs [20]. We identified eight subclasses of NSEs in *P. sibiricum*, including *GALE*, *UGD*, *UGE*, *UGDH*, *UXE*, *RHM*, *UER1*, and *GMDS*. In all, 47 transcripts encoding NSEs were found in this species (Table 6, Table S3). By comparison of the fragments per kilobase of transcript per Million mapped reads (FPKM) from different RNA-Seq libraries, the most abundant transcript was for mannose-1-phosphate guanylyltransferase (*GMPP*), followed by *pgm* (phosphoglucomutase) and *sacA* (β-fructofuranosidase).

Protein encoded by *pgm* is responsible for the production of Glc-1P, using Glc-6P as a precursor. The enzyme encoded by *UGDH* converts UDP-Glu into UDP-GluA, while the enzyme encoded by *GMPP* converts Man-1P into GDP-Man. *SacA* converts sucrose into fructose and Glc-6P. The relatively higher abundance of such transcripts determined here suggests that the biological process of conversion from Glc-6P to Glc-1P should be very active.

### 2.6. Analysis of Differential Gene Expression in Lueyang, Shaanxi (SXLY) and Luoyang, Henan (HNLY) Germplasms

**Figure 5:**
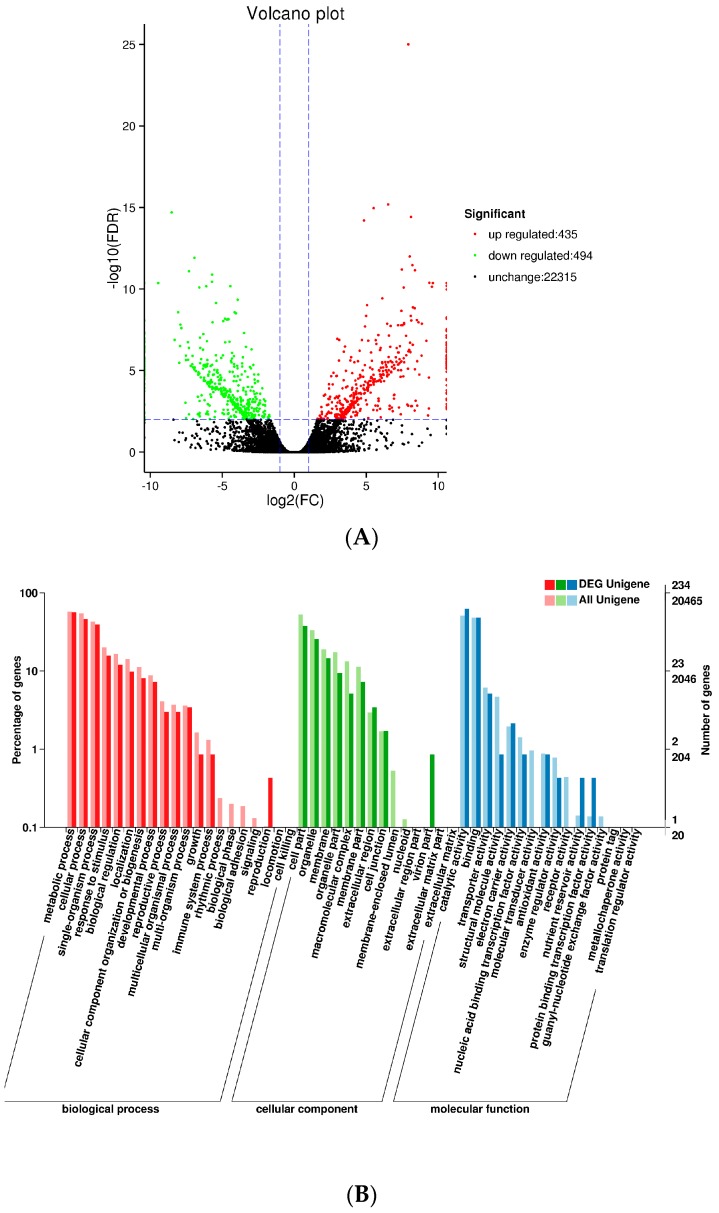
Volcanic map and GO classifications of differentially expressed genes. (**A**) Volcanic plot map of unigenes; (**B**) GO classifications of DEG unigenes. FDR: false discovery rate; DEG: differentially expressed gene.

We used DESeq to analyze differences in gene expression between SXLY and HNLY. In the screening process, the false discovery rate (FDR) was set at <0.01 and the fold change (FC) was >2. The Volcano Plot presented a clear visual of the relationship between the FDR and FC for all genes of interest, enabling us to view expression levels quickly. As shown in Figure 5A, 435 genes were upregulated and 494 were downregulated. These upregulated and downregulated (929) genes were assigned to 50 functional groups (Figure 5B). Among those categorized in “biological processes”, 132 were related to “metabolic process” (GO: 0008152), followed by “cellular process” (GO: 0009987; 108). Within the molecular functions, the assignments were mostly enriched in “catalytic activity” (GO: 0003824; 146) and “binding” (GO: 0005488; 112). The GO terms of cellular components were mainly grouped into “cell part” (GO: 0044464; 88) and “organelle” (GO: 0043231; 60).

**Figure 6:**
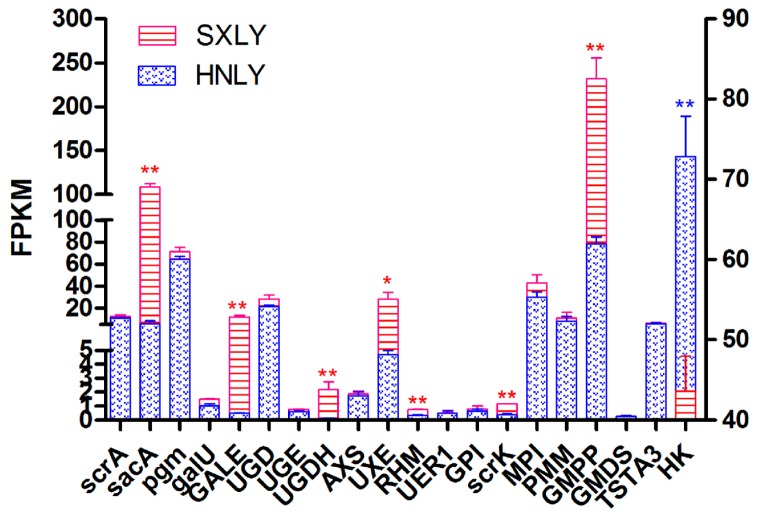
FPKM analysis of candidate unigenes involved in PSP biosynthesis. For a given gene, expression is significantly different between germplasms at * *p* < 0.05 and ** *p* < 0.01. Values on y-axis along right side of figure pertain to *HK*; values along left side are for all other genes.

Among these upregulated and downregulated genes, we focused more on those involved in the pathway of PSP biosynthesis. Based on the abundance of their transcripts, as determined through RNA-seq, we selected eight candidate unigenes: *sacA*, *GALE*, *UGDH*, *UXE*, *RHM*, *scrK*, *GMPP*, and *HK* (Figure 6). Those sharing the least identity with their homologs in the dataset were *scrK* (Unigene 107,236; 53% identity) and *UGDH* (Unigene 153,987; 64% identity). Percent identities for the others—*sacA* (Unigene 157,362), *GALE* (Unigene 51,042), *UXE* (Unigene 153,844), *RHM* (Unigene 142,186), *HK* (Unigene 137,389), and *GMPP* (Unigene 151,473)—ranged from 72% to 99%. Details for all of these alignments are shown in Table S4. This analysis primarily confirmed that our chosen potential unigenes are rather conservative and responsible for PSP biosynthesis.

### 2.7. Analysis of PSP Biosynthetic Pathway

**Figure 7:**
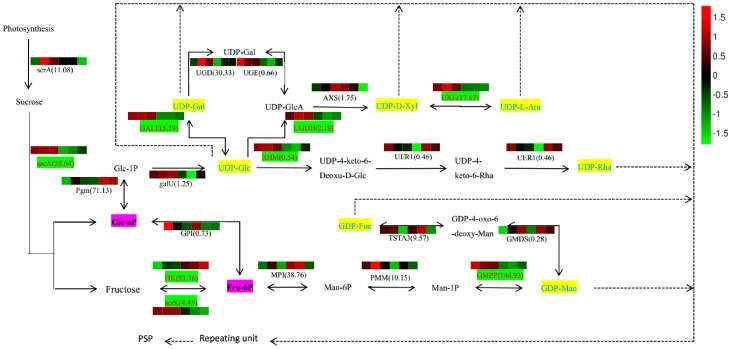
Proposed pathways for polysaccharide biosynthesis in *Polygonatum sibiricum*. Activated monosaccharide units, marked in blue with yellow background; key enzymes, marked in red with green background. Bolded text with pink background indicates key intermediates, while corresponding FPKM values are shown in brackets. Various color blocks represent logarithm of FPKM values. First three colors are SXLY samples; the latter three, HNLY samples. The real line arrows represent the identified enzymatic reactions, and the dashed line arrows represent multiple enzymatic reactions by multiple steps.

Based on our results that identified constituents in the polysaccharide biosynthesis pathway and monosaccharide composition (Table S2), we outlined potential biosynthetic pathways for PSP formation from sucrose (Figure 7). In this scheme, the biosynthesis of PSP comprises three main steps. First, Glc-1P is converted to UDP-Glc immediately, and Fru-6P to GDP-Man indirectly, and then converted in the second step into other NDP-sugars by NSEs. Finally, the NDP-sugars are added to the polysaccharide by several GTs. Various color blocks represent the logarithms of FPKM values for different samples, using normalized data processing and plotting on a heatmap produced with R software. The first three colors in that figure are for SXLY samples; the latter three, for HNLY samples. The key enzymes are highlighted in red and are responsible for the biosynthesis of Glc-6P (*sacA*), Fru-6P (*scrK*), UDP-Gal (*GALE*), UDP-GlcA (*UGDH*), UDP-Ara (*UXE*), UDP-4-keto-6-Deoxu-D-Glc (*RHM*), fructose (*HK*), and GDP-Man (*GMPP*). Whereas expression of *sacA*, *scrK*, *GALE*, *UGDH*, *RHM*, *GMPP*, and *UXE* are positively correlated to the PSP content, that of *HK* is negatively correlated.

**Figure 8:**
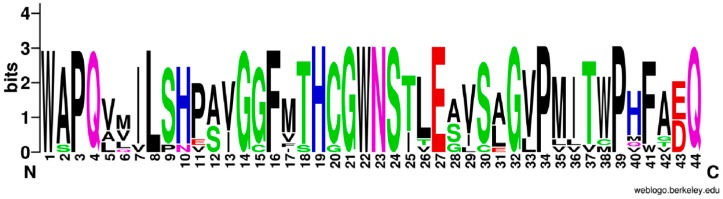
Consensus sequence defining glycosyltransferase plant secondary product glycosyltransferase (PSPG) motif of 18 UDP glycosyltransferases (UGTs) in *P. sibiricum*. The larger font size the more conserved residues.

Various NDP-sugars form a growing polysaccharide chain by the action of glycosyltransferases (GTs) (EC: 2.4.x.y). In the *P. sibiricum* library, we identified 380 unigenes encoding GTs, based on KGG annotations. Among them, 18 unigenes are UDP glycosyltransferases (UGTs) (Table S5). In general, approximately 48% of UGTs have a plant secondary product glycosyltransferase (PSPG) box [22]. This motif contains 44 conserved amino acids. We used WebLogo (http://weblogo.berkeley.edu/logo.cgi) to examine the typical PSPG amino acid sequences for these 18 UGTs. As shown in Figure 8, the font size represents the intensity of the conserved properties for amino acid residues. That is, the 1st and 22nd tryptophan (W); 3rd, 34th, and 39th proline (P); 4th and 44th glutamine (Q); 8th leucine (L); 14th, 21st, and 32nd glycine (G); and 19th histidine (H) are more conserved than any of the other amino acids in the motif. A previous study of the structure-activity relationship in UGTs has shown that the N-terminal domain is the acceptor binding site and the C-terminal domain is the donor response site [23].

### 2.8. Real-Time PCR Analysis

Performing a qRT-PCR analysis enabled us to determine the abundance of transcripts of genes for enzymes related to PSP biosynthesis. After selecting the reference genes in *P. sibiricum* (Table S6), we chose *β-Tubulin* (*TUB*) as our internal reference. We then screened eight genes as candidate unigenes based on the significantly broad differences in their levels of expression between germplasms (Figure 6). Among these candidate unigenes, we predicted that those encoding *sacA*, *scrK*, and *HK* should function upstream of the proposed pathway for PSP biosynthesis (Figure 7), while those encoding *GALE*, *RHM*, *UGDH*, *UXE*, and *GMPP* should act downstream.

**Figure 9:**
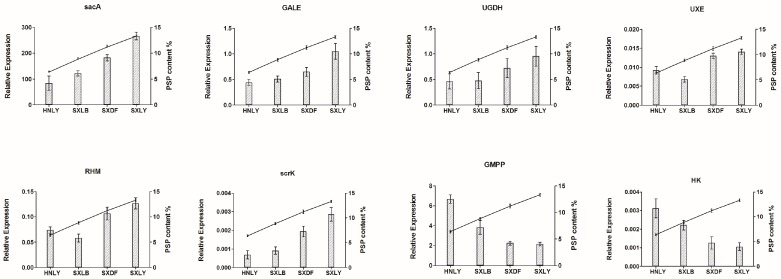
The expression of candidate unigenes involved in PSP biosynthesis between different germplasms. *y*-Axis on left side of each chart indicates the level of relative expression based on results from real-time PCR. Error bars show SD (*n* = 9). *y*-Axis on right side of each chart indicates % PSP content.

We also examined the relative expression of these eight candidate genes in four germplasms: SXLY, HNLY, SXLB, and SXDF (Figure 9). That of *sacA*, *GALE*, *UGDH*, *UXE*, *RHM*, and *scrK* was positively correlated with polysaccharide content, while the relative expression of *GMPP* and *HK* was negatively correlated. By comparing the results of qRT-PCR and RNA-seq, we found that only the expression pattern of *GMPP* was inconsistent between the two results. The enzymes encoded by *GMPP* can convert Man-1P into GDP-Man in both directions. Maybe too much accumulation of PSP can cause *GMPP* negative feedback regulation, leading to the opposite results between the RNA-seq and qRT-PCR.

## 3. Discussion

As far as we know, this is the first time de novo sequencing of *Polygonatum sibiricum* has been conducted that has utilized the Illumina HiSeq 2500 platform. Our sequencing produced 72.06 Gb of clean data and 74,130 unigenes after assembly, making it possible to understand PSP biosynthesis at the molecular level. When compared with datasets published for other medicinal plants, *P. sibiricum* has more unigenes than *Neolamarckia cadamba* (55,432), but fewer than *Erigeron breviscapus* (182,527) and *Gentiana rigescens* (76,717) [24,25,26]. However, the unigenes obtained here average 900.9 bp, which is longer than those from *N. cadamba* (803.2 bp), *E. breviscapus* (738 bp), and *G. rigescens* (757 bp). Notably, the size distribution is homogeneous and 30.36% of the unigenes (22,512) are longer than 1000 bp. This demonstrates a higher quality of assembly results and the reliability of Illumina Hiseq-2500 technology for studying medicinal plants.

Through the analysis of bioinformatics, we identified the unigenes of PSP biosynthesis. The results indicated that sucrose was the precursor of PSP. The real-time PCR results indicated that *GMPP* was more abundant than those of the other candidates. This suggested that, in *P. sibiricum*, this pathway functions as a key branch of PSP biosynthesis. When compared with the molar ratio of monosaccharide composition in PSP biosynthesis (Table S2), we found that Man had a higher ratio (0.478) than that computed for Glc (0.285), Gal (0.139), Ara (0.064), Rha (0.020), and Fuc (0.014). This showed that Man is the most important component in PSP biosynthesis. The enzyme encoded by *GMPP* converts Man-1P into GDP-Man. Our results also revealed that *GMPP* transcripts were the most abundant in the *P. sibiricum* library. It has been reported that reducing the activity of GMPP will seriously affect the accumulation of mannose content, and ultimately destroy the plant leaf cell wall biosynthesis [27]. So, further studies are needed to identify the molecular mechanism that modulates PSP content and monosaccharide composition.

As a key enzyme that functions upstream of PSP biosynthesis, β-fructofuranosidase (encoded by *sacA*) is responsible for the conversion of sucrose to Glc-6P and fructose. Therefore, the high abundance of Unigene 157,362 may be related to the relatively high amount of PSP. The consistent abundance pattern of those transcripts in all tested germplasms confirmed the conservation and importance of this enzyme in PSP production. Studies have shown that the increase of β-fructofuranosidase activity would help improve fructooligosaccharides (FOS) productivity and reduce the time needed for completion of FOS biosynthesis [28]. Future research should focus on elucidating the relationships among transcript abundance, enzyme activity, and PSP content.

Both hexokinase and fructokinase are key enzymes involved in PSP biosynthesis, catalyzing the conversion of fructose to Fru-6P. In tea (*Camellia sinensis*), hexokinase and fructokinase act as important parts in the sugar signaling, carbohydrate metabolism, and response to abiotic stresses. [29]. Although hexokinase (encoded by *HK*) and fructokinase (encoded by *scrK*) function in the same branches of the PSP biosynthesis pathway, they exhibit opposite patterns of expression, thereby showing that they cooperate in that pathway. Additional investigation will enable us to improve our understanding of the mechanism(s) by which PSP biosynthesis and accumulations are regulated at the molecular level.

## 4. Materials and Methods

### 4.1. Ethics Statement

Experimental materials were collected across China, but the field studies did not involve endangered or protected species. This study was conducted at the National Engineering Laboratory for Resource Development of Endangered Chinese Crude Drugs in Northwest China, Xi’an, China.

### 4.2. Plant Materials

Thirteen different germplasms of *Polygonatum sibiricum* were collected throughout China in 2015. The origins of the collected samples are specified in Table S7, and all of the germplasms were authenticated by Prof. Yaping Xiao (Key Laboratory of the Ministry of Education for Medicinal Resources and Natural Pharmaceutical Chemistry, Shaanxi Normal University). The samples were deposited at the National Engineering Laboratory for Resource Development of Endangered Chinese Crude Drugs in Northwest China. For isolating and detecting the polysaccharides, we used rhizomes from these 13 germplasms. Our mRNA-sequencing analysis utilized rhizomes collected from Lueyang, Shaanxi (SXLY) and Luoyang, Henan (HNLY). Each germplasm had three biological repetitions. The germplasm samples were first washed with tap water, then dried on filter paper and cut into small pieces with low temperature before freezing with liquid nitrogen. They were stored at −70 °C prior to library preparation and RNA-sequencing (RNA-seq), which used the Illumina HiSeqTM 2500 system (BioMarker Technologies Co., Ltd, Beijing, China). Validation by qRT-PCR involved not only the SXLY and HNLY germplasms required for transcription, but also rhizomes collected from Danfeng (SXDF) (33°68′24″ N, 110°29′66″ E) and Liuba (SXLB) (33°37′27″ N, 106°54′55″ E), Shaanxi Province. All qRT-PCR were repeated in three biological and three technical replications. These rhizome samples were also washed and stored as described above.

### 4.3. Isolation and Detection of Polysaccharides

The polysaccharides were isolated from *P. sibiricum* rhizomes with a similar method which has been reported earlier [30]. Dried powders (100 g) from rhizomes of individual germplasms were mixed with petroleum ether to remove lipids and then extracted twice at 80 °C (3 h each time) in boiling distilled water (1:30, *w*/*v*). The water extract was collected and concentrated by a rotary evaporator. Afterward, a four-fold volume of 95% ethanol was added. The mixture was held overnight at 4 °C to precipitate the polysaccharide before percolation. Protein was removed from the sediment through repeated freeze-thawing before the mixture was dialyzed and lyophilized to obtain the crude polysaccharide. The weight was recorded and the content of PSP was calculated per 100 g of dry powder.

### 4.4. Library Preparation and Sequencing (mRNA-Seq)

Total RNA was obtained from each sample, using a total RNA Isolation Kit (TIANGEN, Beijing, China) according to the manufacturer’s instructions. Qualified RNAs were used to construct an RNA library for sequencing on the HiSeq 2500 platform.

### 4.5. De Novo Assembly and Functional Annotation

After obtaining high-quality sequencing data, de novo assembly was performed with Trinity software [31]. We then conducted BLAST [32] to compare our unigene sequences with those available from the databases of Non-Redundant (NR) [33]; Swiss-Prot [34]; Gene Ontology (GO) [35]; Clusters of Orthologous Groups (COG) [36]; euKaryotic Orthologous Groups (KOG) [37]; and Kyoto Encyclopedia of Genes and Genomes (KEGG) [38]. We then applied KOBAS 2.0 software [39] to obtain the unigene KEGG orthologies. Using HMMER software (Howard Hughes Medical Institute, San Francisco, CA, USA) [40], we compared our output with the Pfam database [41]. After setting the BLAST E-value parameter at less than 1 × 10^−5^ and the HMMER parameter E-value at less than 1 × 10^−10^, we obtained the unigene annotations. Software for TransDecoder (version 2.0.1, Commonwealth Scientific and Industrial Research Organisation, Canberra, Australia) [42] was also used to predict coding sequences and their corresponding amino acid sequences.

### 4.6. Analysis of Differential Gene Expression

Bowtie software (version 1.1.2, Johns Hopkins University, Baltimore, MD, USA) [43] was used to compare sequenced reads with the unigene database. Based on those results, RSEM [44] was run to estimate expression levels. We then computed the values for Fragments Per Kilobase of transcript per Million mapped reads, or FPKM [45] to determine the abundance of corresponding unigene transcripts. Software for DESeq [46] was used to compare patterns of gene expression between the two germplasm groups, SXLY and HNLY. For purposes of screening, the FDR was set at less than 0.01 and the FC at more than 2.

### 4.7. Identification of Genes Involved in PSP Biosynthesis

Genes involved in PSP biosynthesis were identified based on the KEGG pathway annotation dataset. This included the metabolic pathways for amino sugar, nucleotide sugar, glucose, and starch. Neighbor-Joining (NJ) phylogenetic analysis of homologs for candidate genes was completed with MEGA 6 software. All potential transcripts annotated as glycosyltransferases (GTs) were classified via BLASTx. Then we used WebLogo (http://weblogo.berkeley.edu/logo.cgi) to examine the typical plant secondary product glycosyltransferase (PSPG) amino acid sequences for those UDP glycosyltransferases (UGTs).

### 4.8. Real-Time PCR

Reference genes were selected with geNORM, NormFinder, and BestKeeper. Specific primers were designed by Premier 3.0 [47]. The details of 20 candidate genes and the necessary primers are presented in Table S8. Total RNA was extracted from the SXLY, HNLY, SXDF, and SXLB rhizomes and reverse-transcribed to cDNA with Prime-Script RT Master Mix (TaKaRa, Kyoto, Japan), following the manufacturer’s instructions. Real-time PCR was performed using a 2× Sybr Green qPCR Mix (Aidlab, Beijing, China) and a Light Cycler 96 Instrument (Roche, Germany). Relative expression was calculated by the 2^−ΔΔ*C*t^ method [48,49]. All qRT-PCR were repeated in three biological and three technical replications. The relative expressions were analyzed as means ± standard deviation (SD).

## 5. Conclusions

To summarize, we found that *sacA*, *HK*, and *GMPP* are the key genes that encode enzymes necessary for the PSP metabolic pathway in *Polygonatum sibiricum*. Our results provide new information about PSP biosynthesis in this species at the molecular level. Furthermore, our qRT-PCR analysis of selected unigenes indicated that these candidates function within separate, but equally important, branches of that pathway. These findings should help scientists devise an approach that achieves efficient and sustainable production of PSPs and related bioactive natural products.

## Figures and Tables

**Table 1:** Output statistics from sequencing of *Polygonatum sibiricum* transcriptome.

ID ^1^	Number of Reads	Number of Bases	Guanine-Cytosine (GC) Content	*N* Percentage	% ≥Q30
PSABC	48,703,742	14,514,388,398	50.21%	0.00	93.57
PS01	29,810,908	8,876,080,296	49.81%	0.00	93.40
PS02	30,941,196	9,196,437,754	49.21%	0.00	93.44
PS03	36,392,600	10,740,869,676	49.37%	0.00	93.55
PS04	40,885,773	12,098,862,416	49.70%	0.00	93.31
PS05	22,762,445	6,786,142,412	48.66%	0.00	94.31
PS06	32,982,501	9,849,781,232	48.96%	0.00	93.35

^1^ PSABC: mixed sample of rhizomes, stems, and leaves; PS01-03: rhizomes from Lueyang, Shaanxi (SXLY); PS04-06: rhizomes from Luoyang, Henan (HNLY).

**Table 2:** Output statistics from assembly of *Polygonatum sibiricum* transcriptome sequencing.

Length Range (bp)	Number of Contigs	Transcript Abundance	Number of Unigenes
200–300	23,592,360 (99.41%)	19,417 (14.59%)	15,057 (20.31%)
300–500	72,139 (0.30%)	26,138 (19.63%)	16,631 (22.43%)
500–1000	42,020 (0.18%)	37,052 (27.83%)	19,930 (26.89%)
1000–2000	19,425 (0.08%)	33,660 (25.29%)	15,422 (20.80%)
>2000	7128 (0.03%)	16,854 (12.66%)	7090 (9.56%)
Total number	23,733,072	133,121	74,130
Total length	1,025,259,786	138,445,867	66,783,395
N50 length	45	1532	1364
Mean length	43.20	1040.00	900.90

**Table 3:** Summary statistics of functional annotations for *Polygonatum sibiricum* unigenes via public databases.

Database	Number Annotated	300 bp < Length Number of Unigenes < 1000 bp	Number Longer than 1000 bp
Clusters of Orthologous Groups (COG)	12,175 (16.42%)	4011 (5.41%)	6892 (9.30%)
Gene Ontology (GO)	20,465 (27.61%)	8253 (11.13%)	10,444 (14.09%)
Kyoto Encyclopedia of Genes and Genomes (KEGG)	14,267 (19.25%)	5748 (7.75%)	6797 (9.17%)
euKaryotic Orthologous Groups (KOG)	20,240 (27.30%)	7785 (10.50%)	10,681 (14.41%)
Protein family (Pfam)	24,732 (33.36%)	8700 (11.74%)	14,264 (19.24%)
Swiss-Prot	21,965 (29.63%)	8574 (11.57%)	11,727 (15.82%)
non-redundant (NR)	34,601 (46.68%)	14,135 (19.07%)	17,043 (22.99%)
All annotated	35,793 948.28%)	14,618 (19.72%)	17,140 (23.12%)

**Table 4:** The 20 most-represented KEGG pathways (KO, KEGG Orthology).

Pathway	Number of Unigenes	KO Entry
Ribosome	891 (6.25%)	KO03010
Carbon metabolism	657 (4.61%)	KO01200
Biosynthesis of amino acids	578 (4.05%)	KO01230
Protein processing in endoplasmic reticulum	473 (3.32%)	KO04141
Spliceosome	402 (2.82%)	KO03040
Oxidative phosphorylation	397 (2.78%)	KO00190
RNA transport	365 (2.56%)	KO03013
Glycolysis/Gluconeogenesis	304 (2.13%)	KO00010
Plant hormone signal transduction	295 (2.07%)	KO04075
Starch and sucrose metabolism	284 (1.99%)	KO00500
Plant-pathogen interaction	282 (1.98%)	KO04626
RNA degradation	245 (1.72%)	KO03018
Purine metabolism	243 (1.70%)	KO00230
Carbon fixation in photosynthetic organisms	236 (1.65%)	KO00710
Phagosome	233 (1.63%)	KO04145
Ubiquitin-mediated proteolysis	232 (1.63%)	KO04120
Endocytosis	229 (1.61%)	KO04144
Pyruvate metabolism	227 (1.59%)	KO00620
mRNA surveillance pathway	224 (1.57%)	KO03015
Cysteine and methionine metabolism	215 (1.51%)	KO00270

**Table 5:** The number of unigenes involved in polysaccharide biosynthesis in *Polygonatum sibiricum* database.

Pathway	Number of Unigenes	KO Entry
Glycolysis/Gluconeogenesis	304	KO00010
Starch and sucrose metabolism	284	KO00500
Pyruvate metabolism	227	KO00620
Amino sugar and nucleotide sugar metabolism	215	KO00520
Citrate cycle (Tricarboxylic Acid (TCA) cycle)	170	KO00020
Pentose phosphate pathway	128	KO00030
Galactose metabolism	128	KO00052
Fructose and mannose metabolism	113	KO00051
Pentose and glucuronate interconversions	98	KO00040
*N*-Glycan biosynthesis	74	KO00510
Other glycan degradation	65	KO00511
Glycosylphosphatidylinositol(GPI)-anchor biosynthesis	31	KO00563
Glycosaminoglycan degradation	23	KO00531

**Table 6:** Number of unigenes involved in the biosynthesis of starch and sucrose in *Polygonatum sibiricum*.

Enzyme Code	Enzyme Name	Abbreviation	Number	FPKM
2.7.1.211	Phosphotransferase System	*scrA*	3	11.08
3.2.1.26	β-fructofuranosidase	*sacA*	31	58.64
5.4.2.2	Phosphoglucomutase	*pgm*	5	71.13
2.7.7.9	Uridine-diphosphate glucose pyrophosphorylase	*galU*	8	1.25
5.1.3.2	UDP-glucose 4-epimerase	*GALE*	4	5.59
1.1.1.-	UDP-D-galactose dehydrogenase	*UGD*	4	30.33
5.1.3.6	UDP-glucuronate 4-epimerase	*UGE*	8	0.66
1.1.1.22	UDP-glucose 6-dehydrogenase	*UGDH*	9	2.18
AXS	UDP-apiose/xylose synthase	*AXS*	3	1.75
5.1.3.5	UDP-arabinose 4-epimerase	*UXE*	5	17.67
4.2.1.76	UDP-glucose 4,6-dehydratase	*RHM*	10	0.54
5.1.3.- , 1.1.1.-	3,5-epimerase-4-reductase	*UER1*	4	0.46
5.3.1.9	Glucose-6-phosphate isomerase	*GPI*	7	0.73
2.7.1.1	Hexokinase	*HK*	13	53.76
2.7.1.4	Fructokinase	*scrK*	8	4.45
5.3.1.8	Mannose-6-phosphate isomerase	*MPI*	3	38.76
5.4.2.8	Phosphomannomutase	*PMM*	1	10.15
2.7.7.13	Mannose-1-phosphate guanylyltransferase	*GMPP*	8	144.93
4.2.1.47	GDP-mannose 4,6-dehydratase	*GMDS*	3	0.28
1.1.1.271	GDP-L-fucose synthase	*TSTA3*	1	9.57

FPKM: Fragments Per Kilobase of transcript per Million mapped reads.

## Supplementary Materials

Supplementary materials can be found at www.mdpi.com/1422-0067/18/9/1950/s1.
