# Supplementary material for: De Novo Assembly and Analysis of Polygonatum sibiricum Transcriptome and Identification of Genes Involved in Polysaccharide Biosynthesis
Source: Int J Mol Sci. 2017 Sep 12;18(9):1950. doi: 10.3390/ijms18091950 (PMC5618599; doi:10.3390/ijms18091950)
Supplement: Supplementary file 1 [file ijms-18-01950-s001.pdf]

International Journal of
Molecular Sciences

MDPI

Supplementary Material: *De novo* Assembly and Analysis of *Polygonatum sibiricum* Transcriptome and Identification of Genes Involved in Polysaccharide Biosynthesis

Shiqiang Wang, Bin Wang, Wenping Hua, Junfeng Niu, Kaikai Dang, Yi Qiang and Zhezhi Wang

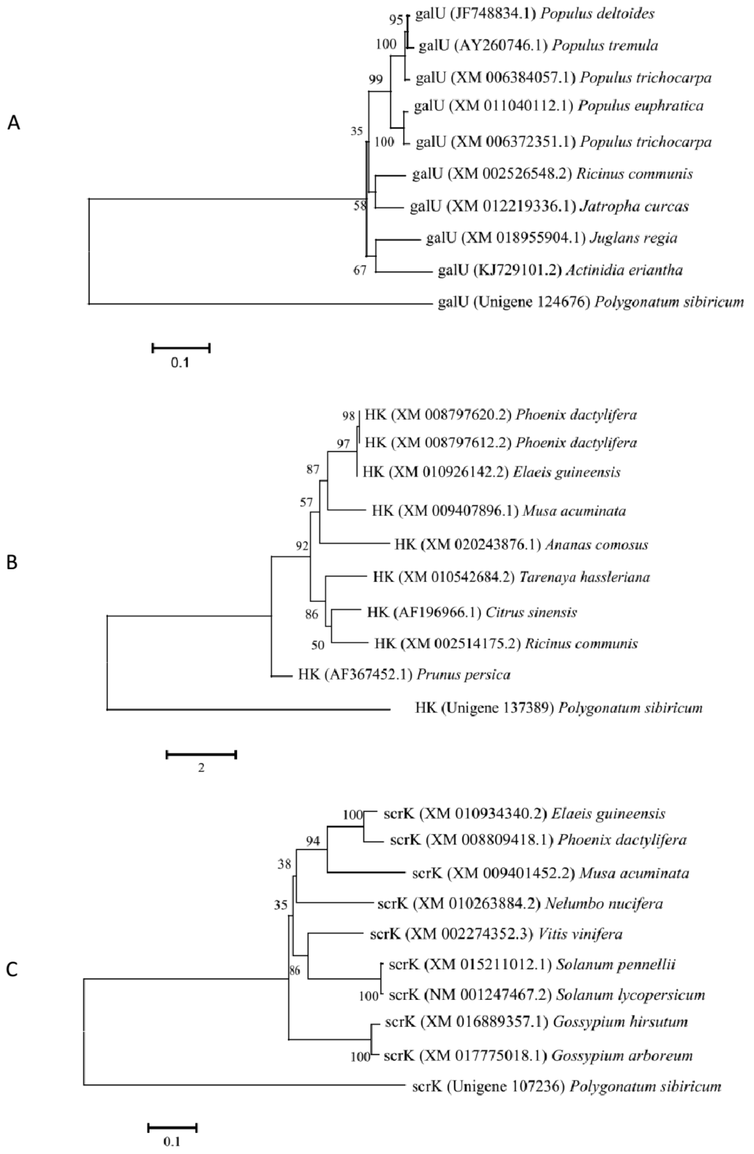

Figure S1. Trees produced from Neighbor-joining phylogenetic analysis of homologs for *galU* (A), *HK* (B), and *scrK* (C) in various plant species, as performed with MEGA 6 software.

PREDICTED: Ricinus communis UTP--glucose-1-phosphate uridylyltransferase (LOC8266519), transcript variant X2, mRNA

Sequence ID: XM_002526548.2 Length: 1889 Number of Matches: 1

Range 1: 777 to 1870 GenBank Graphics ▼ Next Match ▲ Previous Match

Score	Expect	Identities	Gaps	Strand
1151 bits(623)	0.0	953/1111(86%)	28/1111(2%)	Plus/Minus

```
Query  54    TGCCAGAGATTGGGGCTCAAGTTTCCAAATT-TA-TAAGTCTGTAGGAGCTATC---ACA  108
Sbjct  1870  TGCCAGAGATCGAGGCACAAGTTTGCACATTATACTAA-TCTGTAGAAGTTATCCGAACA  1812

Query  109   CTCCAACCTAGTTTTACACCCTATATTATAAAGTTTGTATG-A-ACaaaaaaaCAGGTTC  166
Sbjct  1811  C-CC-ATGTAGTTTTACATCCTCT-TTAT-CAGATTGTATGTATACACAAAAATAATATC  1756

Query  167   A-CCGTATCTAG-GCAGACATTCCAAATTCA-GACTCAGCAAACAAATGCAACTCTCTTA  223
Sbjct  1755  ATAAGTA-GAAGAGC-GGCATTCCGAATTCAAGACACAGGAAAGGGAA-CCACTCTCTTA  1699

Query  224   TTCTCCCAT-AAAAATGTAAGGGCATTCCGAGATGCAGAAAGGACTCCTCAAAGAGCCAC  282
Sbjct  1698  TTCTGCTATCAAAAATACATGAGCATTGTAAGATGGAGAAATGACTCCTCAAAGAG----  1643

Query  283   CACGTCTCCCCCACACTTTTAGCAACCGCTACTTTCTTAGAGGTCTTCAGGACCATTAAT  342
Sbjct  1642  ----TCTCCAACACAATGTCAGCCAA-GCAACTCTTTTACAGGTCCTCAGGGCCATTAAT  1588

Query  343   ATCCTTGTTCACAATTATGGCTCCATCAGGTATTTCCAATTTTACACCAGGTTTTGCAGC  402
Sbjct  1587  ATCCTTGTTCTCAACTACAGCACCATCAGGTATTTCCAATTTCACACCAGGTTGTGCTGA  1528

Query  403   AATGTTGACTTTCCCCTTGAGAGTAATACCAGCACCAAACCACACATCACCAGTCACCTT  462
Sbjct  1527  AATAGTCACTTTCCCCTTGAGTATAACACCAGCACCAAACCATACATCACCAGCCACCTT  1468

Query  463   AAGGCTATCAAGCTCAATGATACTAGGGATCGACTTAAATCGACTCAAGAAGTTTGAAAC  522
Sbjct  1467  AAGGCTATCAAGCTCAATGATGCTTGGGATTGACTTAAATCGACTCAAGAAATTGGCAAC  1408

Query  523   CTTCTTAAATTCTGGGCCCAATTCAATAGAAGGATTTGCAGGATTTTGCCTGGCTTTATT  582
Sbjct  1407  CTTCTTAAATTCAGGACCCAGTTCAACAGAAGGATTTGCAGGATTTTTCCTAGCTTTATT  1348

Query  583   TCGGATAACATAGCCATCCTCCAAGATGTAAAGATCAGACTGGACAAGAAGCAAATCTGA  642
Sbjct  1347  TCTGATAACAAAGCCACCGTCTAAGGTGTAAAGATCAGACTGGACAAGAAGCAAATCTGA  1288

Query  643   AGTTGCCTTCACTGGAAGGAATCGGGATCGAGGAACATTTATGCCAATTGCATGGTCAAA  702
Sbjct  1287  GGTTGCCTTCACTGGAAGGAATCGAGATCGAGGAACATTTATACCAATTGCATGATCAAA  1228

Query  703   GAACCTAATAGCGGCACCAGCTGCAGTTTCTAGTTGAAGAACTTTGACTCCATCTACTTC  762
Sbjct  1227  AAACCTAATAGCTGCACCAGCCGCAGTTTCAAGCTGAAGAACTTTGACGCCATCCACTTC  1168

Query  763   CTTTGGGTTAGGAATTATCTCCATCTTTAGAGCATCAGCTTCCACCAGCCTTTTAATTGC  822
Sbjct  1167  CTTTGGGTTGGGAATTATCTCCATCTTTAATGCATCAGCTTCCACAAGCCTTTTAATTGC  1108

Query  823   TGACAAGCTCACCCACAAGTTGTTTGTATTGAAAATTTTGAACTTCTCTATTGATTTGAA  882
Sbjct  1107  TGACAAGCTCACCCACAAATTGTTTGTATTGAAAATTTTGAACTTCTCTATTGACTTGAA  1048

Query  883   TTCACTGACATGCTCATCAGGGACTTGCGCAATCTCCAACAGCTGAACCTTCCCTTCATA  942
Sbjct  1047  TTCATTGACATGCTCATCAGGGACTTGTGCAATTTCCAATAACTGAACCTTCCCTTCGTA  988

Query  943   GGAGATGAGAGTACCGCCCTTTACATCAGCCAGGGTTTTGGGTGTCACCTCCATGCAGTA  1002
Sbjct  987   AGAGATGAGAGTGCCACCCTTCACATCAGCCAGGGTTTTGGGTGTCACCTCCATGCAGTA  928

Query  1003  TTCATTCTTGTTTCTGATTAGATGACTTAAGATTTTCAAATCAACTATAGCACCAAGGTT  1062
Sbjct  927   TTCATTCTTGTTTCTGATCAGATGATTTAAAATTTTCAAATCAACAATAGCACCAAGGTT  868

Query  1063  ATCTGAGTTGGCAATAAATACGTACTCCTTGCCCTGTAATAACAAAGAATCAAGCTTGCC  1122
Sbjct  867   ATCTGAGTTGGCAGCAAACACATACTCCTTGCCCTGAGATAACAGAGCATCAAGCTTGCC  808

Query  1123  ACTGTTCTTCAAGGATGGGAACACATCACCA  1153
Sbjct  807   ACTGTTCCTCAAGGATGGGAACACATCACCA  777
```

Figure S2. Alignment of *galU* between *Polygonatum sibiricum* and *Ricinus communis*.

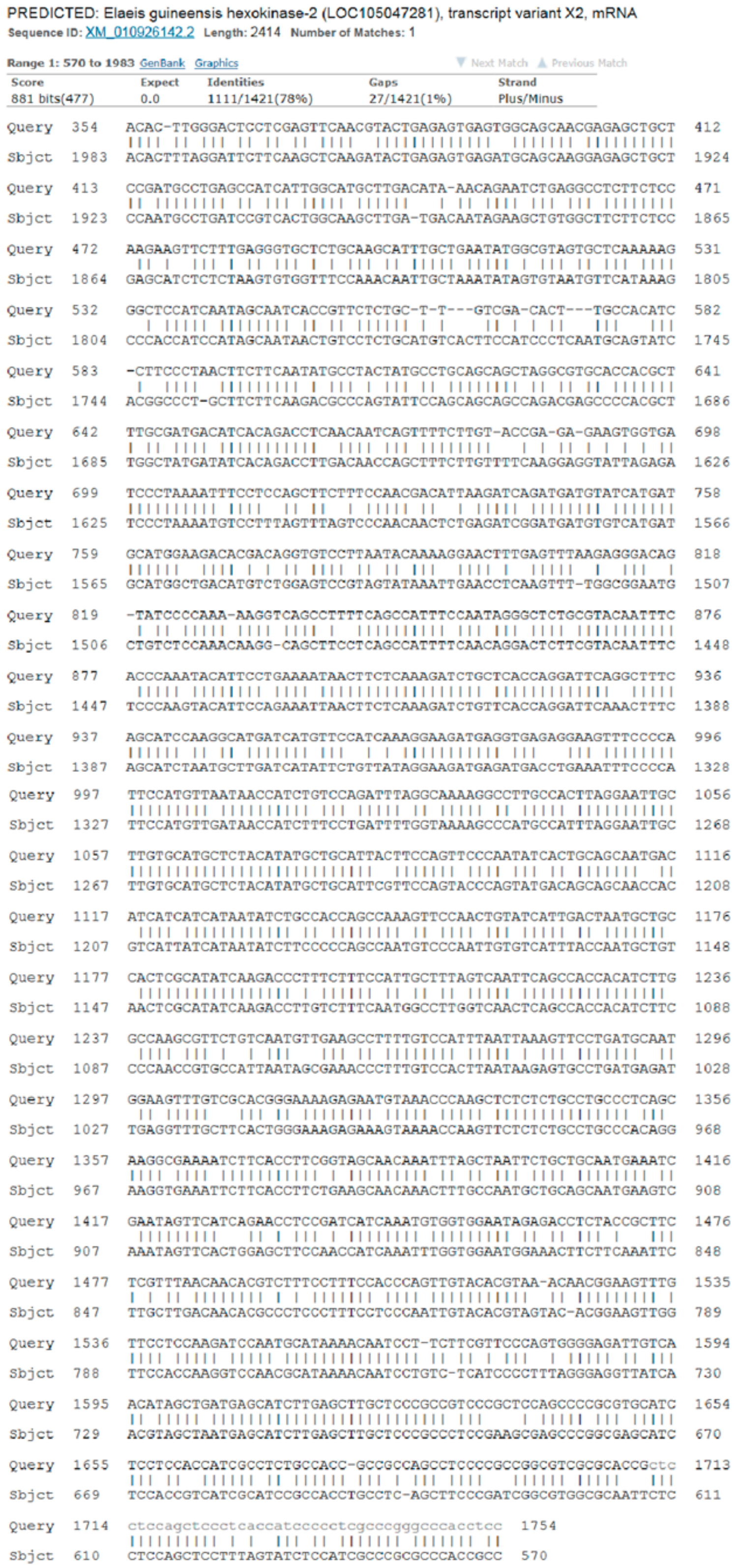

Figure S3. Alignment of *HK* between *Polygonatum sibiricum* and *Phoenix dactylifera*.

PREDICTED: Elaeis guineensis probable fructokinase-6, chloroplastic (LOC105053244), mRNA

Sequence ID: XM_010934340.2 Length: 1496 Number of Matches: 1

Range 1: 223 to 1179 GenBank Graphics ▼ Next Match ▲ Previous Match

Score	Expect	Identities	Gaps	Strand
992 bits(537)	0.0	817/957(85%)	0/957(0%)	Plus/Minus

```
Query  384   AGCAACTACATTAACCAGAGCATCGAGCACAGCTTGTCGCGTTGGAAGAGCTGGTATGGC  443
             |||||||| ||||||||  ||||  | ||| || | ||| ||||| | ||| || || ||
Sbjct  1179  AGCAACTATATTAACCAAGGCATTCATCACCGCATCTCGAGTTGGTAAAGCAGGGATTGC  1120

Query  444   TCCTCTCTCCATCACTGTCAATGCTCCGCATGCATTTGCAAACTTGAGAGCATCTGAAAG  503
             ||||||||||||||| |||| ||| || ||||||||||||||| | || || |||   ||
Sbjct  1119  TCCTCTCTCCATCACGGTCAGTGCACCACATGCATTTGCAAACCTAAGGGCTTCTACTAG  1060

Query  504   TCGACCTTCATCCTGAAGCAAAGAAATATCACCAGCTAATTGAGATAATATTCCAGCAAC  563
             ||||||||||||||| ||||| |||||||||   |||||||| ||||| |||||||||||
Sbjct  1059  TCGACCTTCATCCTGGAGCAAGGAAATATCAGTGGCTAATTGCGATAAAATTCCAGCAAC  1000

Query  564   AAAAGCATCCCCAGCGCCAGTCGTGTCTACTTGATTCACCTTTAAACCACTAACCCTCCC  623
             ||||||||||||||| ||||| ||||| ||   |||||| ||||  ||||| ||||||||
Sbjct  999   AAAAGCATCCCCAGCACCAGTAGTGTCCACCGCATTCACTTTTAGTCCACTTACCCTCCC  940

Query  624   ATGGAATTCCTTAGTATAATATCTGCAGCCATCTGGGCCCTCAGTGACGAGAAGCAACTT  683
             |  ||||||||| ||||||||||||||||| | ||||||||||||||| |||||||||||
Sbjct  939   ACTGAATTCCTTGGTATAATATCTGCAGCCCTGTGGGCCCTCAGTGACAAGAAGCAACTT  880

Query  684   CAGATTTGGATGGAAAAGCTTACGGACAACATTATCATCATAAGGATCTTCACCTTTAGT  743
              |||||||| ||||| ||||| || |||||   ||||||||| |||||||| ||||||||
Sbjct  879   AAGATTTGGGTGGAAGAGCTTGCGTACAACTGCATCATCATATGGATCTTCTCCTTTAGT  820

Query  744   TAAAAAGGAGATTTCTTCTTCACTTATCTTGATTATATCAGCAAAGTCCCATATGCTTAA  803
              |||||||| ||||||||||||||||||||||| |||||||||   ||||||||||| ||
Sbjct  819   CAAAAAGGAAATTTCTTCTTCACTTATCTTGATAATATCAGCAGCATCCCATATGCTCAA  760

Query  804   GATTCCATCTCTAGCACTCTCAGCAGAAGGCCATAGCGGAAGTCGTAGATTTGGATCATA  863
             ||||||||||||||||||||| ||||| |||||||| ||||| |  ||||||||||||||
Sbjct  759   GATTCCATCTCTAGCACTCTCTGCAGATGGCCATAGTGGAAGCCTCAGATTTGGATCATA  700

Query  864   TGACAGAAGAACTCCAGCATCCTTAGCAGCTTTAGCTGCTGCAATATGTGCTGACTTGCA  923
              || ||||| |||||||||||||||||| |  |||||||||||||||||||||| || ||
Sbjct  699   AGAAAGAAGCACTCCAGCATCCTTAGCAACCATAGCTGCTGCAATATGTGCTGATTTACA  640

Query  924   TGGTTCTGTGATCAGACTTATAGATCCATAGTGAAATATTTTTGCCTTCTTTATGATATC  983
             |||||| ||||| |||||||||||||||||||| || || ||||||||| | ||||| ||
Sbjct  639   TGGTTCGGTGATAAGACTTATAGATCCATAGTGGAAGATCTTTGCCTTCCTAATGATGTC  580

Query  984   CAGTTCAAGTTCTTCTTCTTGAAGAAGCATATCGGCACTGGGATTACGATAAAACATGAA  1043
              || ||||||||||  ||||  || |||||||| ||||||||||||||||||||||||||
Sbjct  579   AAGGTCAAGTTCTTTCTCTTCGAGCAGCATATCAGCACTGGGATTACGATAAAACATGAA  520

Query  1044  TTCACGTTCACCATCACTTCTCAACGTGACAAAAGCCAAAGCTGTTCTCGCACCAGGATC  1103
              ||||||||||||||| ||||||| || |||||||||||||||||||  ||||| |||||
Sbjct  519   CTCACGTTCACCATCATTTCTCAATGTAACAAAAGCCAAAGCTGTTCGAGCACCCGGATC  460

Query  1104  AAAACGCATTCCTTCGTTGTTCACATTATTTTTCTTTAAAATGTCAGCCAGCATATAGCC  1163
              || ||||||||||  ||||||||||||||||  |||||||| ||||| |||||||| ||
Sbjct  459   GAACCGCATTCCTTGATTGTTCACATTATTTTCTTTTAAAATATCAGCTAGCATATACCC  400

Query  1164  AAACTCATCTTCACCAACCTTCCCAATGAAGGCAGCTGAACCACCGAGCCGAGCAATTCC  1223
             ||||||||||||||||||||||||||| ||||| | ||||||||| || ||||| || ||
Sbjct  399   AAACTCATCTTCACCAACCTTCCCAATAAAGGCTGATGAACCACCAAGACGAGCTATGCC  340

Query  1224  AACGGCAACATTGGCCGGTGCTCCCCCAGGAGCCTTCTTGAACGCAGGGGCCTCAGCCAA  1283
             ||| |||||||| ||||||||||| ||||||||||||||||| || || |  ||| | ||
Sbjct  339   AACAGCAACATTAGCCGGTGCTCCTCCAGGAGCCTTCTTGAAAGCTGGAGATTCACCTAA  280

Query  1284  CGACAGCCCACTAATCGTCGGAACAAAGTCGATCAGCATCTCCCCAAAGCACACGAC  1340
              || |  ||||||||||| || ||||| || || ||||| |||||||| ||||||||
Sbjct  279   TGATAAGCCACTAATCGTGGGACAAAATCAATTAGCATTTCCCCAAAACACACGAC  223
```

Figure S4. Alignment of scrK between *Polygonatum sibiricum* and *Elaeis guineensis*.

Table S1. Number of unigenes from *Polygonatum sibiricum* assigned to KEGG reference pathways.

Pathway	Pathway_ID	Number of unigenes
Ribosome	KO03010	891
Carbon metabolism	KO01200	657
Biosynthesis of amino acids	KO01230	578
Protein processing in endoplasmic reticulum	KO04141	473
Spliceosome	KO03040	402
Oxidative phosphorylation	KO00190	397
RNA transport	KO03013	365
Glycolysis/Gluconeogenesis	KO00010	304
Plant hormone signal transduction	KO04075	295
Starch and sucrose metabolism	KO00500	284
Plant–pathogen interaction	KO04626	282
RNA degradation	KO03018	245
Purine metabolism	KO00230	243
Carbon fixation in photosynthetic organisms	KO00710	236
Phagosome	KO04145	233
Ubiquitin-mediated proteolysis	KO04120	232
Endocytosis	KO04144	229
Pyruvate metabolism	KO00620	227
mRNA surveillance pathway	KO03015	224
Cysteine and methionine metabolism	KO00270	215
Amino sugar and nucleotide sugar metabolism	KO00520	215
Phenylpropanoid biosynthesis	KO00940	206
Ribosome biogenesis in eukaryotes	KO03008	199
Glyoxylate and dicarboxylate metabolism	KO00630	176
Citrate cycle (TCA cycle)	KO00020	170
Pyrimidine metabolism	KO00240	170
Glutathione metabolism	KO00480	169
Peroxisome	KO04146	165
Fatty acid metabolism	KO01212	160
Arginine and proline metabolism	KO00330	152
Glycine, serine and threonine metabolism	KO00260	149
Phenylalanine metabolism	KO00360	149
2-Oxocarboxylic acid metabolism	KO01210	136
Photosynthesis	KO00195	135
Proteasome	KO03050	133
Pentose phosphate pathway	KO00030	128
Galactose metabolism	KO00052	128
Glycerophospholipid metabolism	KO00564	119
Fructose and mannose metabolism	KO00051	113
Alanine, aspartate and glutamate metabolism	KO00250	111
Aminoacyl-tRNA biosynthesis	KO00970	110
Fatty acid degradation	KO00071	107
Inositol phosphate metabolism	KO00562	103
alpha-Linolenic acid metabolism	KO00592	100
Pentose and glucuronate interconversions	KO00040	98
Glycerolipid metabolism	KO00561	98
Terpenoid backbone biosynthesis	KO00900	98

Valine, leucine and isoleucine degradation	KO00280	97
Phosphatidylinositol signaling system	KO04070	96
Phenylalanine, tyrosine and tryptophan biosynthesis	KO00400	91
Ascorbate and aldarate metabolism	KO00053	88
Nucleotide excision repair	KO03420	85
Protein export	KO03060	81
Cyanoamino acid metabolism	KO00460	80
beta-Alanine metabolism	KO00410	78
Biosynthesis of unsaturated fatty acids	KO01040	77
Fatty acid biosynthesis	KO00061	76
Circadian rhythm – plant	KO04712	76
Porphyrin and chlorophyll metabolism	KO00860	75
N-Glycan biosynthesis	KO00510	74
Tyrosine metabolism	KO00350	71
Regulation of autophagy	KO04140	70
Sulfur metabolism	KO00920	69
Lysine degradation	KO00310	68
Propanoate metabolism	KO00640	67
DNA replication	KO03030	66
Ubiquinone and other terpenoid-quinone biosynthesis	KO00130	65
Other glycan degradation	KO00511	65
Flavonoid biosynthesis	KO00941	64
Homologous recombination	KO03440	63
Sphingolipid metabolism	KO00600	61
Basal transcription factors	KO03022	61
Photosynthesis - antenna proteins	KO00196	60
Nitrogen metabolism	KO00910	59
ABC transporters	KO02010	59
Steroid biosynthesis	KO00100	56
Base excision repair	KO03410	56
SNARE interactions in vesicular transport	KO04130	56
RNA polymerase	KO03020	55
Tryptophan metabolism	KO00380	54
Mismatch repair	KO03430	53
One carbon pool by folate	KO00670	49
Pantothenate and CoA biosynthesis	KO00770	49
Selenocompound metabolism	KO00450	47
Valine, leucine and isoleucine biosynthesis	KO00290	46
Carotenoid biosynthesis	KO00906	44
Fatty acid elongation	KO00062	41
Tropane, piperidine and pyridine alkaloid biosynthesis	KO00960	39
Ether lipid metabolism	KO00565	38
Histidine metabolism	KO00340	37
Butanoate metabolism	KO00650	37
Biotin metabolism	KO00780	32
Glycosylphosphatidylinositol(GPI)-anchor biosynthesis	KO00563	31
Degradation of aromatic compounds	KO01220	31
Cutin, suberine and wax biosynthesis	KO00073	30

Linoleic acid metabolism	KO00591	30
Thiamine metabolism	KO00730	29
Folate biosynthesis	KO00790	29
Stilbenoid, diarylheptanoid and gingerol biosynthesis	KO00945	29
Arachidonic acid metabolism	KO00590	28
Lysine biosynthesis	KO00300	27
Diterpenoid biosynthesis	KO00904	25
Isoquinoline alkaloid biosynthesis	KO00950	24
Glycosaminoglycan degradation	KO00531	23
Vitamin B6 metabolism	KO00750	23
Zeatin biosynthesis	KO00908	23
Nicotinate and nicotinamide metabolism	KO00760	22
Brassinosteroid biosynthesis	KO00905	21
C5-Branched dibasic acid metabolism	KO00660	18
Taurine and hypotaurine metabolism	KO00430	17
Glycosphingolipid biosynthesis - globo series	KO00603	17
Sulfur relay system	KO04122	17
Riboflavin metabolism	KO00740	16
Non-homologous end-joining	KO03450	15
Other types of O-glycan biosynthesis	KO00514	13
Monoterpenoid biosynthesis	KO00902	12
Sesquiterpenoid and triterpenoid biosynthesis	KO00909	12
Glycosphingolipid biosynthesis - ganglio series	KO00604	11
Limonene and pinene degradation	KO00903	11
Caffeine metabolism	KO00232	9
Flavone and flavonol biosynthesis	KO00944	8
Synthesis and degradation of ketone bodies	KO00072	7
Lipoic acid metabolism	KO00785	7
Vancomycin resistance	KO01502	6
Betalain biosynthesis	KO00965	2
Glucosinolate biosynthesis	KO00966	2
Polyketide sugar unit biosynthesis	KO00523	1
Anthocyanin biosynthesis	KO00942	1

Table S2. Monosaccharide composition of PSP.

Monosaccharide species	RT (min)	Start time	End time	Area	Area %	Height	Height %	A/H	Mole ratio
Ara	18.342	18.167	18.517	3507611	6.36	601690	11.62	5.83	0.064
Man	28.077	27.942	28.175	26357768	47.81	5142115	19.81	5.13	0.478
Gal	28.266	28.167	28.492	15724869	28.52	3293556	17.41	4.77	0.285
Glc	28.884	28.775	29.042	7688552	13.95	1413024	31.27	5.44	0.139
Rha	17.656	17.542	17.792	1076870	1.95	185790	13.34	5.80	0.020
Fuc	18.319	18.008	18.217	772584	1.40	130211	6.55	5.93	0.014

Table S3. Functional annotations of unigenes identified as NDP-sugar interconversion enzymes (NSEs) in *Polygonatum sibiricum*.

Enzyme abbreviation	Gene_ID	KO丨e_value丨Database_Genes丨Anno
GALE	c51042.graph_c0	K01784丨0丨pda:103714167丨bifunctional UDP-glucose 4-epimerase and UDP-xylose 4-epimerase 1; K01784 UDP-glucose 4-epimerase [EC:5.1.3.2] (A)
	c71739.graph_c0	K01784丨3.23118e-42丨sly:101248179丨UDP-glucose 4-epimerase GEPI48-like; K01784 UDP-glucose 4-epimerase [EC:5.1.3.2] (A)
	c103420.graph_c0	K01784丨1.4068e-167丨cmo:103482842丨UDP-glucose 4-epimerase GEPI48; K01784 UDP-glucose 4-epimerase [EC:5.1.3.2] (A)
	c161496.graph_c0	K01784丨4.81375e-171丨tcc:TCM_016202丨UDP-D-glucose/UDP-D-galactose 4-epimerase 1 isoform 1; K01784 UDP-glucose 4-epimerase [EC:5.1.3.2] (A)
UGD	c143337.graph_c0	K12451丨1.23939e-36丨pmum:103337384丨probable rhamnose biosynthetic enzyme 1; K12451 3,5-epimerase/4-reductase [EC:5.1.3.- 1.1.1.-] (A)
	c143337.graph_c1	K12451丨1.38416e-160丨fve:101295808丨probable rhamnose biosynthetic enzyme 1-like; K12451 3,5-epimerase/4-reductase [EC:5.1.3.- 1.1.1.-] (A)
	c144506.graph_c0	K18121丨1.04555e-167丨mus:103995976丨glyoxylate/succinic semialdehyde reductase 1; K18121 glyoxylate/succinic semialdehyde reductase [EC:1.1.1.79 1.1.1.-] (A)
	c151030.graph_c0	K18121丨5.3521e-95丨mus:103992702丨glyoxylate/succinic semialdehyde reductase 2, chloroplastic isoform X1; K18121 glyoxylate/succinic semialdehyde reductase [EC:1.1.1.79 1.1.1.-] (A)
UGE	c170719.graph_c0	K08679丨1.34076e-09丨pda:103701187丨UDP-glucuronate 4-epimerase 1-like; K08679 UDP-glucuronate 4-epimerase [EC:5.1.3.6] (A)
	c94561.graph_c0	K08679丨1.49726e-12丨vvi:100241904丨UDP-glucuronate 4-epimerase 6; K08679 UDP-glucuronate 4-epimerase [EC:5.1.3.6] (A)
	c132132.graph_c0	K08679丨0丨vvi:100241904丨UDP-glucuronate 4-epimerase 6; K08679 UDP-glucuronate 4-epimerase [EC:5.1.3.6] (A)
	c153724.graph_c0	K08679丨0丨pvu:PHAVU_003G152300g丨hypothetical protein; K08679 UDP-glucuronate 4-epimerase [EC:5.1.3.6] (A)
	c154937.graph_c0	K08679丨0丨pda:103701187丨UDP-glucuronate 4-epimerase 1-like; K08679 UDP-glucuronate 4-epimerase [EC:5.1.3.6] (A)
	c161016.graph_c1	K08679丨0丨pda:103710561丨UDP-glucuronate 4-epimerase 3-like; K08679 UDP-glucuronate 4-epimerase [EC:5.1.3.6] (A)
	c161619.graph_c0	K08679丨0丨vvi:100241904丨UDP-glucuronate 4-epimerase 6; K08679 UDP-glucuronate 4-epimerase [EC:5.1.3.6] (A)
	c110330.graph_c0	K08679丨0丨rcu:RCOM_0782170丨UDP-glucuronate 5-epimerase, putative (EC:5.1.3.12); K08679 UDP-glucuronate 4-epimerase [EC:5.1.3.6] (A)
UGDH	c168782.graph_c0	K00012丨2.98471e-61丨mtr:MTR_7g012950丨UDP-glucose dehydrogenase; K00012 UDP-glucose 6-dehydrogenase [EC:1.1.1.22] (A)
	c169751.graph_c0	K00012丨1.16617e-110丨pmum:103330088丨UDP-glucose 6-dehydrogenase 1-like; K00012 UDP-glucose 6-dehydrogenase [EC:1.1.1.22] (A)
	c171350.graph_c0	K00012丨7.26368e-97丨cmo:103492904丨UDP-glucose 6-dehydrogenase 1; K00012 UDP-glucose 6-dehydrogenase [EC:1.1.1.22] (A)
	c85329.graph_c0	K00012丨3.2189e-12丨cme:CYME_CMB031C丨UDP-glucose 6-dehydrogenase; K00012 UDP-glucose 6-dehydrogenase [EC:1.1.1.22] (A)
	c137637.graph_c0	K00012丨7.88132e-130丨ccp:CHC_T00008869001丨UDP-glucose dehydrogenase; K00012 UDP-glucose 6-dehydrogenase [EC:1.1.1.22] (A)
	c104353.graph_c0	K00012丨4.36344e-73丨pda:103695952丨UDP-glucose 6-dehydrogenase 5; K00012 UDP-glucose 6-dehydrogenase [EC:1.1.1.22] (A)
	c153987.graph_c0	K00012丨0丨mus:103981423丨UDP-glucose 6-dehydrogenase 4; K00012 UDP-glucose 6-dehydrogenase [EC:1.1.1.22] (A)
	c156202.graph_c0	K00012丨0丨mus:103980185丨UDP-glucose 6-dehydrogenase 4; K00012 UDP-glucose 6-dehydrogenase [EC:1.1.1.22] (A)
	c109773.graph_c0	K00012丨7.5067e-31丨pda:103718180丨UDP-glucose 6-dehydrogenase 5-like; K00012 UDP-glucose 6-dehydrogenase [EC:1.1.1.22] (A)

UXE	c70998.graph_c0	K12448|1.97108e-171|rcu:RCOM_0771330|UDP-glucose 4-epimerase, putative (EC:5.1.3.5); K12448 UDP-arabinose 4-epimerase [EC:5.1.3.5] (A)
	c90008.graph_c0	K12448|1.09507e-36|mus:103979020|probable UDP-arabinose 4-epimerase 2 isoform X1; K12448 UDP-arabinose 4-epimerase [EC:5.1.3.5] (A)
	c126582.graph_c0	K12448|0|pxb:103952320|UDP-arabinose 4-epimerase 1-like; K12448 UDP-arabinose 4-epimerase [EC:5.1.3.5] (A)
	c152282.graph_c0	K12448|0|vvi:100259390|UDP-arabinose 4-epimerase 1; K12448 UDP-arabinose 4-epimerase [EC:5.1.3.5] (A)
	c153844.graph_c0	K12448|0|pda:103720550|probable UDP-arabinose 4-epimerase 3; K12448 UDP-arabinose 4-epimerase [EC:5.1.3.5] (A)
RHM	c169139.graph_c0	K12450|5.78399e-13|cit:102619504|probable rhamnose biosynthetic enzyme 1-like; K12450 UDP-glucose 4,6-dehydratase [EC:4.2.1.76] (A)
	c180306.graph_c0	K12450|4.12276e-13|gsl:Gasu_48500|dTDP-glucose 4,6-dehydratase (EC:4.2.1.46); K12450 UDP-glucose 4,6-dehydratase [EC:4.2.1.76] (A)
	c90376.graph_c0	K12450|4.4872e-59|bdi:100829812|probable rhamnose biosynthetic enzyme 1; K12450 UDP-glucose 4,6-dehydratase [EC:4.2.1.76] (A)
	c132526.graph_c0	K12450|1.49864e-72|csv:101223284|probable rhamnose biosynthetic enzyme 2-like; K12450 UDP-glucose 4,6-dehydratase [EC:4.2.1.76] (A)
	c134353.graph_c0	K12450|0|pda:103708634|probable rhamnose biosynthetic enzyme 1; K12450 UDP-glucose 4,6-dehydratase [EC:4.2.1.76] (A)
	c139404.graph_c0	K12450|0|gmx:100785885|probable rhamnose biosynthetic enzyme 1-like; K12450 UDP-glucose 4,6-dehydratase [EC:4.2.1.76] (A)
	c142186.graph_c0	K12450|0|pop:POPTR_0001s39210g|hypothetical protein; K12450 UDP-glucose 4,6-dehydratase [EC:4.2.1.76] (A)
	c147565.graph_c0	K12450|3.9146e-90|aly:ARALYDRAFT_478892|hypothetical protein; K12450 UDP-glucose 4,6-dehydratase [EC:4.2.1.76] (A)
	c109180.graph_c0	K12450|1.82623e-153|mus:103980241|probable rhamnose biosynthetic enzyme 1; K12450 UDP-glucose 4,6-dehydratase [EC:4.2.1.76] (A)
	c166824.graph_c0	K12450|0|vvi:100260586|trifunctional UDP-glucose 4,6-dehydratase/UDP-4-keto-6-deoxy-D-glucose 3,5-epimerase/UDP-4-keto-L-rhamnose-reductase RHM1; K12450 UDP-glucose 4,6-dehydratase [EC:4.2.1.76] (A)
UER1	c174959.graph_c0	K12451|4.18211e-43|tcc:TCM_016154|Nucleotide-rhamnose synthase/epimerase-reductase; K12451 3,5-epimerase/4-reductase [EC:5.1.3.- 1.1.1.-] (A)
	c120308.graph_c0	K12451|0|rcu:RCOM_1683000|NAD dependent epimerase/dehydratase, putative; K12451 3,5-epimerase/4-reductase [EC:5.1.3.- 1.1.1.-] (A)
	c143337.graph_c0	K12451|1.23939e-36|pmum:103337384|probable rhamnose biosynthetic enzyme 1; K12451 3,5-epimerase/4-reductase [EC:5.1.3.- 1.1.1.-] (A)
	c143337.graph_c1	K12451|1.38416e-160|fve:101295808|probable rhamnose biosynthetic enzyme 1-like; K12451 3,5-epimerase/4-reductase [EC:5.1.3.- 1.1.1.-] (A)
GMDS	c69282.graph_c0	K01711|7.57744e-65|csv:101208005|GDP-mannose 4,6 dehydratase 2-like; K01711 GDP-mannose 4,6-dehydratase [EC:4.2.1.47] (A)
	c86965.graph_c0	K01711|4.79449e-24|mus:103970338|GDP-mannose 4,6 dehydratase 1-like; K01711 GDP-mannose 4,6-dehydratase [EC:4.2.1.47] (A)
	c128821.graph_c0	K01711|1.22005e-141|cit:102613014|GDP-mannose 4,6 dehydratase 2-like; K01711 GDP-mannose 4,6-dehydratase [EC:4.2.1.47] (A)

Table S4. Identities of candidate unigenes related to PSP biosynthesis, analyzed at nucleotide level. Sequence similarities with each specified gene from *Polygonatum sibiricum* (% Identity in last column) were calculated by the Basic Local Alignment Search Tool (BLAST) (http://blast.ncbi.nlm.nih.gov/).

A) *sacA*

Gene	Species	Accession number	Max score	Total score	Query cover	E-value	Identity
AtsacA1	*Agave tequilana*	JN790054.1	883	883	77%	0.0	77%
AtsacA2	*A. tequilana*	DQ535031.1	880	880	77%	0.0	77%
AtsacA3	*A. tequilana*	JN790053.1	869	869	77%	0.0	77%
AtsacA4	*A. tequilana*	EU026119.2	399	399	45%	1e-106	75%
AtsacA5	*A. tequilana*	JN790060.1	239	443	37%	2e-58	76%

B) *GALE*

Gene	Species	Accession number	Max score	Total score	Query cover	E-value	Identity
PdGALE	*Phoenix dactylifera*	XM_008801334.2	815	815	77%	0.0	81%
MaGALE	*Musa acuminate*	XM_009414368.2	749	749	78%	0.0	80%
OlGALE	*Ornithogalum longebracteatum*	KU664038.1	652	652	78%	0.0	78%
PbGALE	*Pyrus x bretschneideri*	XM_009347175.2	545	545	76%	8e-151	77%
FvGALE	*Fragaria vesca*	XM_004298698.2	540	540	76%	4e-149	77%
AiGALE	*Arachis ipaensis*	XR_001618403.1	538	538	76%	1e-148	76%

C) *UGDH*

Gene	Species	Accession number	Max score	Total score	Query cover	E-value	Identity
EgUGDH	*Elaeis guineensis*	XM_010938031.2	998	998	64%	0.0	83%
BdUGDH	*Brachypodium distachyon*	XM_003577751.3	998	998	64%	0.0	83%
BoUGDH	*Bambusa oldhamii*	AY773130.1	985	985	64%	0.0	83%
SiUGDH	*Setaria italica*	XM_004981649.3	953	953	64%	0.0	82%
PdUGDH	*Phoenix dactylifera*	XM_008787743.2	944	944	65%	0.0	82%
OsUGDH	*Oryza sativa*	XM_015764669.1	937	937	64%	0.0	82%
AtUGDH	*Aegilops tauschii*	XM_020331265.1	926	926	64%	0.0	82%
CeUGDH	*Colocasia esculenta*	AY222335.1	920	920	64%	0.0	82%

D) *UXE*

Gene	Species	Accession number	Max score	Total score	Query cover	E-value	Identity
PdUXE	*Phoenix dactylifera*	XM_008810308.2	896	896	82%	0.0	82%
EgUXE	*Elaeis guineensis*	XM_019851960.1	867	867	80%	0.0	82%
MaUXE	*Musa acuminata*	XM_009395015.2	800	800	81%	0.0	81%
AcUXE	*Ananas comosus*	XM_020240417.1	732	732	84%	0.0	80%
ObUXE	*Oryza brachyantha*	XM_006657370.2	649	649	69%	0.0	80%

E) *RHM*

Gene	Species	Accession number	Max score	Total score	Query cover	E-value	Identity
JcRHM	*Jatropha curcas*	XM_012225546.1	2451	2451	99%	0.0	90%
PeRHM	*Populus euphratica*	XM_011008510.1	2109	2109	99%	0.0	87%
JrRHM	*Juglans regia*	XM_018993306.1	1881	1881	99%	0.0	85%
TcRHM	*Theobroma cacao*	XM_007021946.2	1836	1836	99%	0.0	84%
VvRHM	*Vitis vinifera*	XM_002285598.3	1810	1810	99%	0.0	84%
CsRHM	*Citrus sinensis*	XM_006477756.2	1790	1790	100%	0.0	84%
PmRHM	*Prunus mume*	XM_016793132.1	1760	1760	99%	0.0	84%
PbRHM	*Pyrus x bretschneideri*	XM_009378452.2	1688	1688	99%	0.0	83%

F) *scrK*

Gene	Species	Accession number	Max score	Total score	Query cover	E-value	Identity
EgscrK	*Elaeis guineensis*	XM_010934340.2	992	992	53%	0.0	85%
PdscrK	*Phoenix dactylifera*	XM_008809418.1	937	937	53%	0.0	84%
NnscrK	*Nelumbo nucifera*	XM_010263884.2	817	817	53%	0.0	82%
MascrK	*Musa acuminata*	XM_009401452.2	815	815	52%	0.0	82%
VvscrK	*Vitis vinifera*	XM_002274352.3	749	749	50%	0.0	82%
PtscrK	*Populus trichocarpa*	XM_002305164.2	754	754	51%	0.0	81%

G) *GMPP*

Gene	Species	Accession number	Max score	Total score	Query cover	E-value	Identity
PdGMPP	*Phoenix dactylifera*	XM_008804913.2	1070	1070	72%	0.0	84%
EgGMPP	*Elaeis guineensis*	XM_010939248.2	1059	1059	72%	0.0	84%
AcGMPP	*Ananas comosus*	XM_020252994.1	870	870	72%	0.0	81%
ObGMPP	*Oryza brachyantha*	XM_015835653.1	806	806	72%	0.0	80%
MnGMPP	*Morus notabilis*	XM_010096733.1	798	798	72%	0.0	80%
VvGMPP	*Vitis vinifera*	XM_002282386.3	793	793	72%	0.0	80%

H) *HK*

Gene	Species	Accession number	Max score	Total score	Query cover	E-value	Identity
EgHK	*Elaeis guineensis*	XM_010926142.2	881	881	69%	0.0	88%
PdHK	*Phoenix dactylifera*	XM_008797620.2	845	845	72%	0.0	77%
MaHK	*Musa acuminata*	XM_009407896.1	732	732	66%	0.0	77%
CsHK	*Citrus sinensis*	NM_001288857.1	612	612	61%	1e-170	76%
AcHK	*Ananas comosus*	XM_020243876.1	597	597	72%	4e-166	74%
RcHK	*Ricinus communis*	XM_002514175.2	436	436	54%	8e-118	74%
ThHK	*Tarenaya hassleriana*	XM_010542684.2	398	398	71%	4e-106	72%

Table S5. UDP-glycosyltransferases found in *Polygonatum sibiricum*.

Gene_ID	KO|e_value|Database_Genes|Anno
c169792.graph_c0	K13496|0|sly:101257246|UDP-glycosyltransferase 73C1-like; K13496 UDP-glucosyltransferase 73C [EC:2.4.1.-] (A)
c170030.graph_c0	K13679|0|tcc:TCM_015612|UDP-glycosyltransferase superfamily protein isoform 1; K13679 granule-bound starch synthase [EC:2.4.1.242] (A)
c172989.graph_c0	K13679|9.18547e-141|tcc:TCM_015612|UDP-glycosyltransferase superfamily protein isoform 1; K13679 granule-bound starch synthase [EC:2.4.1.242] (A)
c1875.graph_c0	K13691|6.33653e-84|cit:102619890|UDP-glycosyltransferase 74F2-like; K13691 pathogen-inducible salicylic acid glucosyltransferase [EC:2.4.1.-] (A)
c56959.graph_c0	K13496|1.10568e-100|pda:103708808|UDP-glycosyltransferase 73C6-like; K13496 UDP-glucosyltransferase 73C [EC:2.4.1.-] (A)
c6752.graph_c0	K13496|5.76012e-38|gmx:100790358|UDP-glycosyltransferase 73C3-like; K13496 UDP-glucosyltransferase 73C [EC:2.4.1.-] (A)
c70862.graph_c0	K08237|1.3681e-56|sita:101764556|UDP-glycosyltransferase 72B3-like; K08237 hydroquinone glucosyltransferase [EC:2.4.1.218] (A)
c78024.graph_c0	K13496|0|pda:103708833|UDP-glycosyltransferase 73C6-like; K13496 UDP-glucosyltransferase 73C [EC:2.4.1.-] (A)
c85443.graph_c0	K08237|5.1431e-25|tcc:TCM_005182|UDP-glycosyltransferase superfamily protein; K08237 hydroquinone glucosyltransferase [EC:2.4.1.218] (A)
c90528.graph_c0	K13496|3.17133e-144|pda:103708808|UDP-glycosyltransferase 73C6-like; K13496 UDP-glucosyltransferase 73C [EC:2.4.1.-] (A)
c96476.graph_c0	K13679|0|tcc:TCM_015612|UDP-glycosyltransferase superfamily protein isoform 1; K13679 granule-bound starch synthase [EC:2.4.1.242] (A)
c118861.graph_c0	K13496|1.49414e-20|pda:103708833|UDP-glycosyltransferase 73C6-like; K13496 UDP-glucosyltransferase 73C [EC:2.4.1.-] (A)
c130195.graph_c0	K13496|0|pda:103708834|UDP-glycosyltransferase 73C6-like; K13496 UDP-glucosyl transferase 73C [EC:2.4.1.-] (A)
c132414.graph_c0	K13496|4.15324e-43|pda:103708808|UDP-glycosyltransferase 73C6-like; K13496 UDP-glucosyl transferase 73C [EC:2.4.1.-] (A)
c139423.graph_c0	K13496|0|pda:103717036|UDP-glycosyltransferase 73C6-like; K13496 UDP-glucosyl transferase 73C [EC:2.4.1.-] (A)
c146981.graph_c0	K13496|9.06307e-44|mus:103969971|UDP-glycosyltransferase 73C6-like; K13496 UDP-glucosyl transferase 73C [EC:2.4.1.-] (A)
c161382.graph_c1	K13496|0|pda:103708808|UDP-glycosyltransferase 73C6-like; K13496 UDP-glucosyl transferase 73C [EC:2.4.1.-] (A)
c167564.graph_c0	K09480|1.05443e-39|tcc:TCM_022931|UDP-Glycosyltransferase superfamily protein isoform 1; K09480 digalactosyldiacylglycerol synthase [EC:2.4.1.241] (A)

Table S6. Primary selection of reliable internal control for analyzing gene expression in different tissues from *Polygonatum sibiricum*.

Gene symbol	Gene name	Primer sequence (5'-3')	Product size (bp)	PCR efficiency (%)	Mean expression stability (Rank by geNorm)	Stability (Rank by NormFinder software)	CV±SD (Rank by BestKeeper software)
18S	18S rRNA	F:TCAAGCTTCTCTCCCACCAG<br>R:TGGTCTCCTCGGTTCTATGC	227	98.5	0.49	0.19	3.12±0.66
ACT	actin,other eukaryote	F:AGTACTGTTCCAACCGTCGT<br>R:GCGCAGTAATCTCCTTGCTC	182	99.2	0.96	1.04	5.50±1.21
CYP	peptidyl-	F:TCGCTGATGACCACCTTCTT	221	98.8	0.69	0.29	4.59±1.05

	prolyl cis-trans isomerase B	R:TTCAAGTTGGTGCATGTGGG					
GAPDH	glyceraldehyde 3-phosphate dehydrogenase	F:CCATGGTCAACGTCGGAATC R:GCGAAGATGTGGATGGCTTT	229	96.7	0.56	0.21	2.50±0.65
TUA2	tubulin alpha	F:TGAGGAGGTTGGTGCTGAAT R:AGAGAGAGAATCAGGCAGCC	219	97.7	1.61	1.20	8.29±2.55
TUB1	tubulin beta	F:ATCTGGCCAAAAGGACCTGA R:GGTGCTAAGTTCTGGGAGGT	203	99.4	0.41	0.09	1.38±0.36
UBQ10	small subunit ribosomal protein S27Ae	F:AGCTCTGACACCATCGACAA R:AATGGTGTCACTGCTCTCCA	243	97.6	1.25	0.37	5.13±1.35
UBQ5	ubiquitin C	F:CAAGAAGGTCAAGCTCGCTG R:ACCGCTACAACAGATCAAGC	204	96.3	0.52	0.12	2.06±0.76

Table S7. Origins of rhizome samples collected from different germplasms of *Polygonatum sibiricum*.

Origin	Abbreviation	Longitude	Latitude	Elevation (m)
Xiakou Yi, ShaanXi, China	SXXKY	33°12′31″	106°25′32″	909
Shangluo, ShaanXi	SXSL	34°02′18″	109°81′18″	1044
ZhenAn, ShaanXi	SXZA	33°48′84″	109°02′02″	1086
Danfeng, ShaanXi	SXDF	33°68′24″	110°29′66″	953
Liuba, ShaanXi	SXLB	33°37′27″	106°54′55″	1288
Lueyang, ShaanXi	SXLY	33°17′56″	105°51′12″	1192
Foping, ShaanXi	SXFP	33°52′86″	108°01′71″	1494
Luoyang, HeNan, China	HNLY	34°39′41″	112°24′7″	1193
Anyang, HeNan	HNAY	33°30′00″	112°43′50″	820
Yingshan, HuBei, China	HBYS	30°73′79″	115°69′55″	291
Shiyan, HuBei	HBSY	32°45′46″	111°11′54″	705
Tiantai, ZheJiang, China	ZJTT	29°12′83″	120°98′97″	426
Jiangxian, ShanXi, China	SXJX	111°40′38″	35° 27′1″	1085

Table S8. *Polygonatum sibiricum* genes and primers used in real-time PCR.

Gene ID	Candidate gene*	Forward (5' -3')	Tm (°C)	Reverse (5' -3')
c143943.graph_c0	*scrA*	CTTACATGGCCACGAATGTG	59.90	CACACCCGCCAGTTTATGAT
c157362.graph_c0	*sacA*	GGCCGATGTCACCTACAACT	60.00	CTATTTCTCCGGCCTTGGAT
c152224.graph_c0	*Pgm*	CCCAGATTCCATCCTTCTCA	60.00	TTCTGGACTAAAGGGCGTTG
c124676.graph_c0	*galU*	CAGCTTCCACCAGCCTTTTA	60.38	ATGGAGGTGACACCCAAAAC
c51042.graph_c0	*GALE*	TTGCAAGGCTGCTATGTGAC	60.02	TGTGGGAGCTCATGAAAGTG
c143337.graph_c1	*UGD*	ACTATCACCTTCGCCTGCTC	59.46	GACATCCCCAACTCCATGAC
c110330.graph_c0	*UGE*	AAGGCTGTGTCGGTTCTTTG	60.29	AATGGTACGTCACCGTTTCC
c153987.graph_c0	*UGDH*	GCACAAACATAGCCCAAACA	59.59	CTTCAAACTGCATCCTGCAA
c140053.graph_c0	*AXS*	CCTTCCGGAGAGGACTGTCT	60.78	ATCAATCTGGCGGCTATCTG
c153844.graph_c0	*UXE*	AGCAATCTCCTGGTTTTGGA	59.67	AAAAAGCTCTTGCAGCTTCG

c142186.graph_c0	*RHM*	TAAACATGGCCAACCTCTCC	59.93	ATAACGACCCGTGGAAACAA
c120308.graph_c0	*UER1*	GGTTCCTCTTTGCCATCTCA	60.20	CAAAGCCATGGTGGAAGAGT
c149264.graph_c0	*GPI*	GGCCATTTTTGGTGATGAAT	59.63	ATGGGATGGACAAGCACTTC
c137389.graph_c0	*HK*	TCCTCATCCTCCACCTAACG	60.07	CAAGAAAACGACGAACACCA
c107236.graph_c0	*scrK*	AACGCTGCACTTTCCAGTTT	59.92	TCCTCCTCTCGTCAGTGGAT
c157674.graph_c0	*MPI*	GGCTTATGGTTTGGGTCCTT	60.19	ACGCTGAAGTCGTGGGTTAG
c134455.graph_c0	*PMM*	CCAGAGGACACTGCAGAACA	60.02	AAGGATCGCCGGTCTTTTAT
c151473.graph_c0	*GMPP*	TGGTTCTACCTGGGTTCTGG	59.96	CTGGTCCAATCGCAACATC
c128821.graph_c0	*GMDS*	GTGGGTTTGAACTGGAAGGA	59.94	TAACCAGCATCAACGAGCAC
c149334.graph_c0	*TSTA3*	GGTGGAGGAGGAAGAAGGAC	60.05	TCTCTCCAATGGCAGACTCC

**scrA*, Phosphotransferase System; *sacA*, beta-fructofuranosidase; *pgm*, phosphoglucomutase; *galU*, uridine-diphosphate glucose pyrophosphorylase; *GALE*, UDP-glucose 4-epimerase; *UGD*, UDP-D-galactose dehydrogenase; *UGE*, UDP-glucuronate 4-epimerase; *UGDH*, UDP-glucose 6-dehydrogenase; *AXS*, UDP-apiose/xylose synthase; *UXE*, UDP-arabinose 4-epimerase; *RHM*, UDP-glucose 4,6-dehydratase; *UER1*, 3,5-epimerase/4-reductase; *GPI*, glucose-6-phosphate isomerase; *HK*, hexokinase; *scrK*, fructokinase; *MPI*, mannose-6-phosphate isomerase; *PMM*, phosphomannomutase; *GMPP*, mannose-1-phosphate guanylyltransferase; *GMDS*, GDP-mannose 4,6-dehydratase; *TSTA3*, GDP-L-fucose synthase
